# Supplementary material for: Mutant Huntingtin stalls ribosomes and represses protein synthesis in a cellular model of Huntington disease
Source: Nat Commun. 2021 Mar 5;12:1461. doi: 10.1038/s41467-021-21637-y (PMC7935949; doi:10.1038/s41467-021-21637-y)
Supplement: Supplementary file 1 — Supplementary Information [file 41467_2021_21637_MOESM1_ESM.pdf]

Supplementary Information

Mutant Huntingtin Stalls Ribosomes and Represses Protein Synthesis in a Cellular Model of Huntington disease

Mehdi Eshraghi[1], Pabalu Karunadharma[2], Juliana Blin[3], Neelam Shahani[1], Emiliano P. Ricci[3], Audrey Michel[4], Nicolai T. Urban[5], Nicole Galli[1], Manish Sharma[1], Uri Nimrod Ramírez-Jarquín[1], Katie Florescu[1] Jennifer Hernandez[1], and Srinivasa Subramaniam[1, *]

1. The Scripps Research Institute, Department of Neuroscience, Jupiter, FL, USA
2. The Scripps Research Institute, Genomic Core, Jupiter, FL, USA
3. Laboratory of Biology and Cellular Modelling at Ecole Normale Supérieure of Lyon, RNA Metabolism in Immunity and Infection Lab, LBMC, Lyon, France
4. RiboMaps Ltd., Cork, Ireland
5. The Max Planck Neuroscience Institute, Jupiter, FL, USA

*Correspondence ssubrama@scripps.edu

Fig. S1

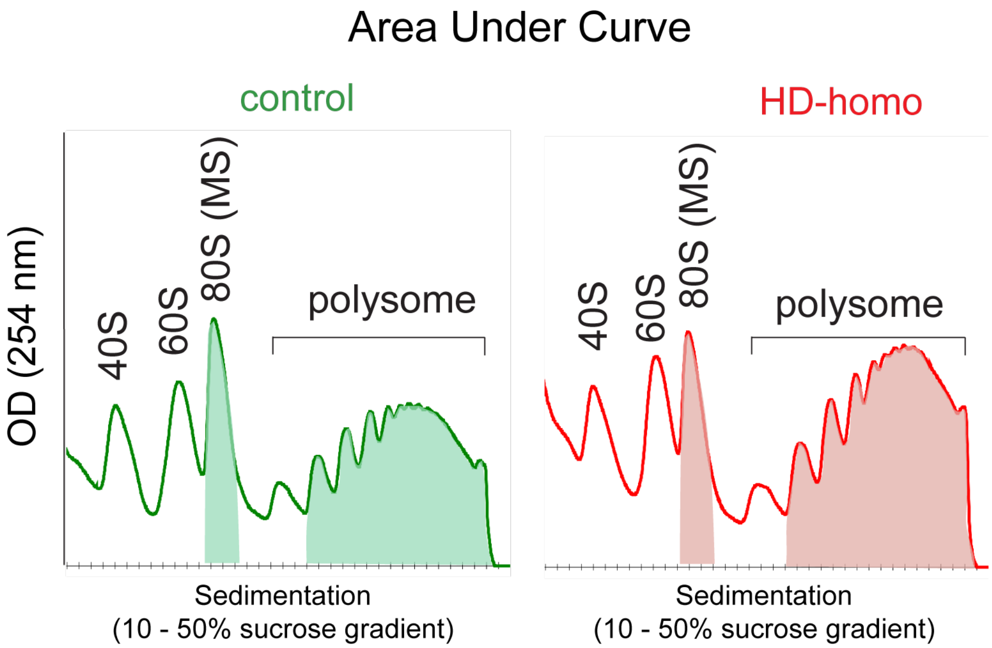

Fig. S1. Area under curve used to calculate PS/MS. Shaded regions indicates the area used to calculate the percentage area under the curve for polysome (PS) and 80S monosome (MS) peaks in control and HD-homo cells, using PeakChart (v. 2.08, Brandel), and expressed as a ratio of PS/MS.

Fig. S2

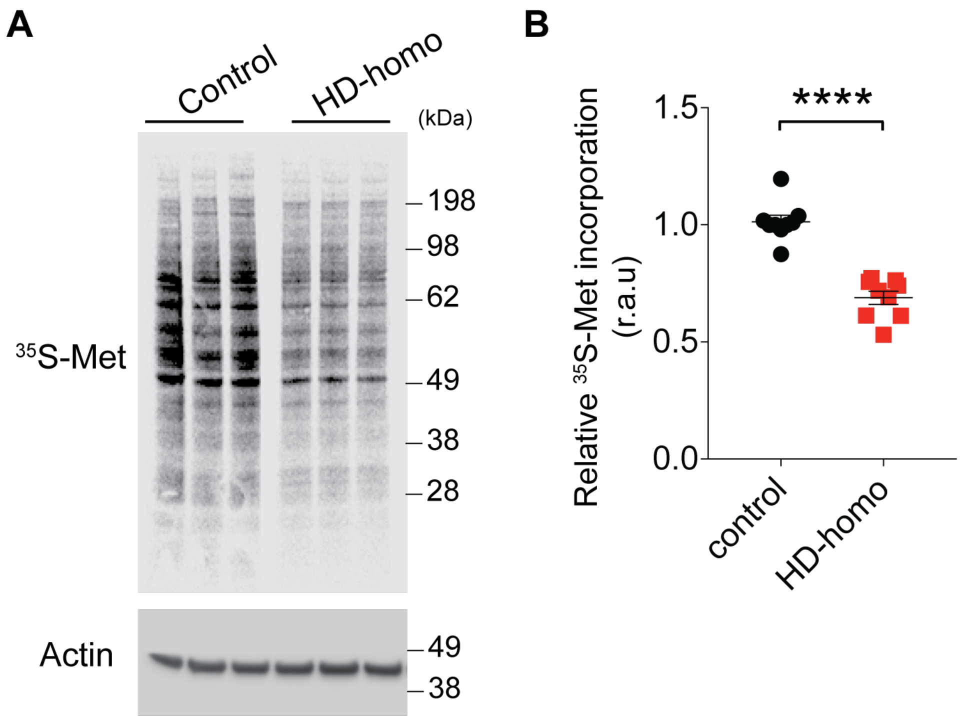

Fig. S2. Protein synthesis is diminished in HD striatal cells. (**A**) Representative autoradiograph of ^{35}S-Met incorporation in control and HD-homo striatal cells, and its quantification in (**B**). The relative arbitrary unit, r. a. u. Data are mean ± SEM (n = 9 independent experiments), ****p < 0.01 two-tailed Student's t-test. Exact p values are reported in the Source Data file. Source data are provided as a Source Data file.

Fig. S3

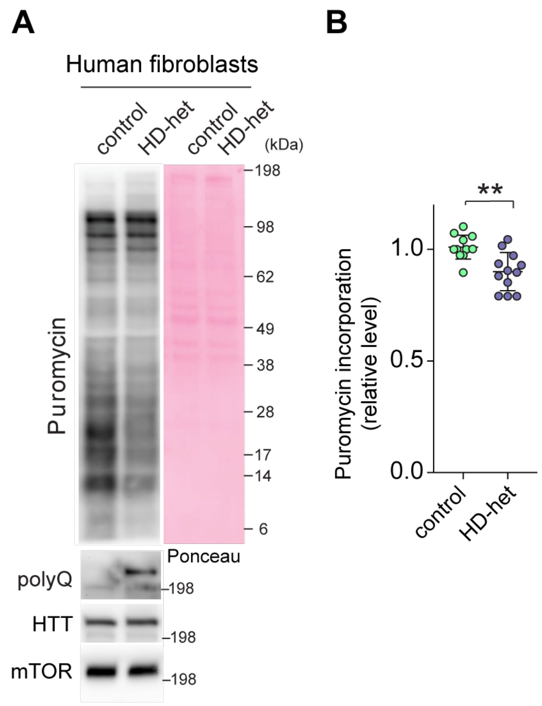

Fig. S3. Protein synthesis is diminished in human HD fibroblasts. (**A**) Puromycin metabolic labeling in human fibroblasts and (**B**) its quantification. Data are mean ± SEM (n= 12, independent experiments), ***p* < 0.01 by two-tailed Student's *t* test. Exact p values are reported in the Source Data file. Source data are provided as a Source Data file.

Fig. S4

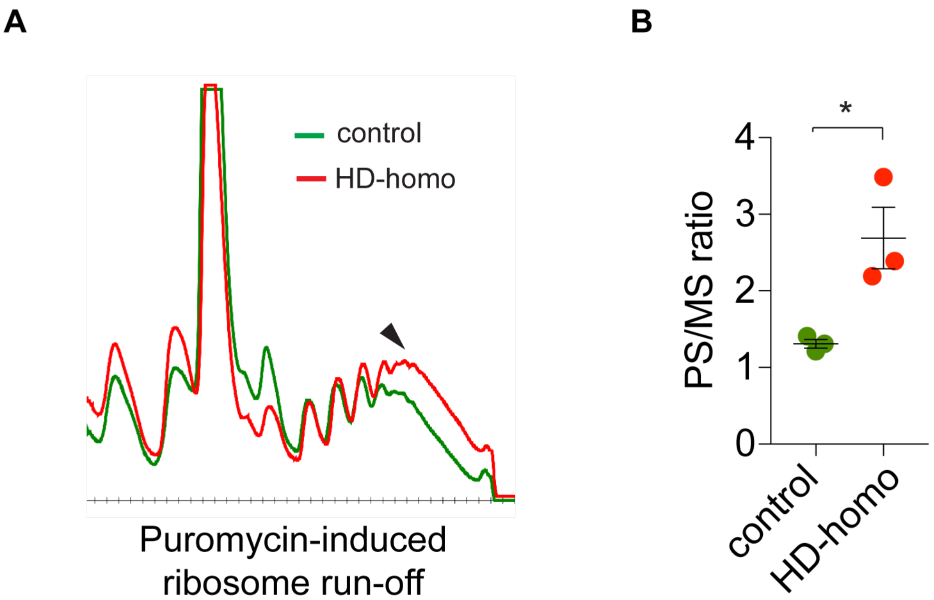

Fig. S4. **Ribosome runoff experiment show slower running of ribosomes in HD**. (**A**) Puromycin (100 μg/ml, 20 min)-induced ribosome-run off in control and HD-homo striatal cells and (**B**) Quantification of PS/MS ratio as measured by the area under the curve for polysome (PS) and 80S monosome (MS) peaks. Data are mean ± SEM (n= 3, independent experiments), $*p < 0.05$ by two-tailed Student's *t* test. Exact p values are reported in the Source Data file. Source data are provided as a Source Data file.

Fig. S5

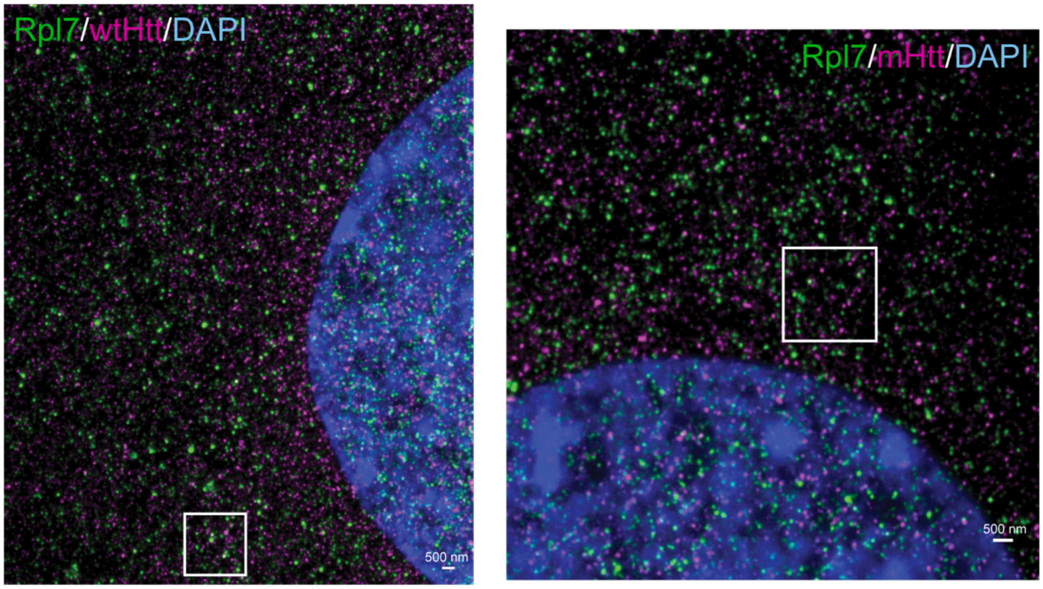

Fig. S5. **Htt and ribosomal protein interaction**. Representative STED image showing localization of Rpl7 (green)/Htt (magenta) or Rpl7 (green)/mHtt (magenta) in control and HD-homo cells using immunocytochemistry technique. Inset indicate the areas shown in main figure 6B. n = 4 for control cells, n = 3 for HD-homo cells. DAPI, nuclear stain.

Fig. S6

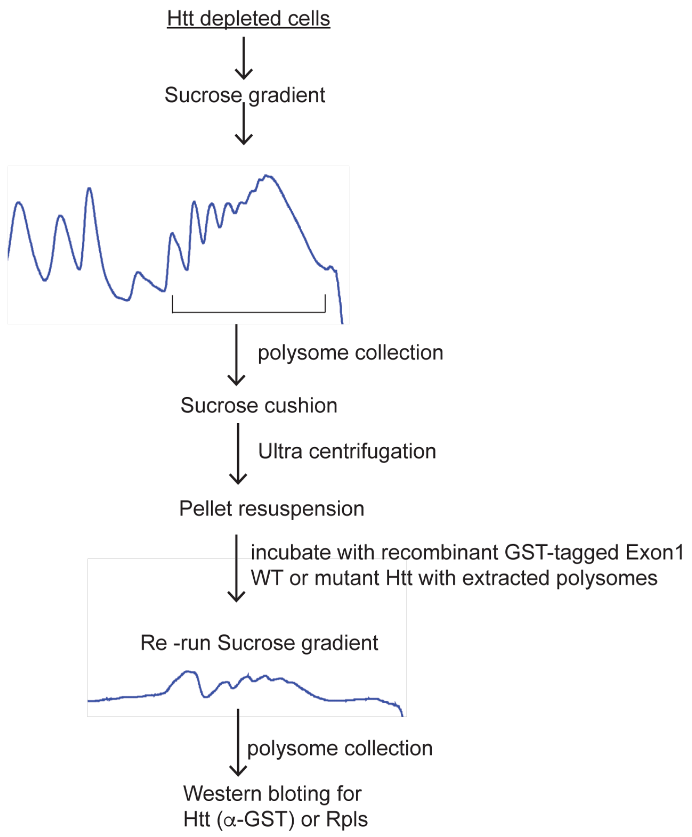

Fig. S6. **Flow chart**. Experimental plan for mHtt in vitro ribosome binding assay in CRISPR-Htt depleted control striatal cells.

Fig. S7

A

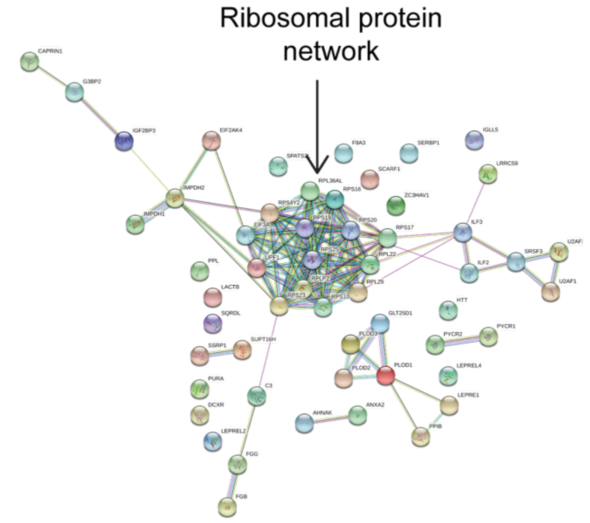

B

Top Biological Process	*p-value*	*molecule*
Non-sense mediated decay	1.61E-10	11
SRP-dependent cotranslation	1.05E-09	10
Protein complex disassembly	1.20E-08	11
Translational initation	1.24E-08	11
Translational termination	1.95E-08	11
Translational elongation	3.90E-08	10
**Top Functions and Components**	*p-value*	*molecule*
RNA binding	2.81E-19	32
poly(A) RNA binding	3.22E-19	29
Structural constituent of ribosome	3.70E-11	11
Cytosolic ribosome	1.14E-14	12
Ribonucleoprotein complex	3.78E-14	20
Ribosomal subunits	4.85E-10	10

Fig. S7. **HTT forms complex with ribosome proteins**. STRING/IPA analysis found translation and ribosome/RNA binding components (**A**), and an enrichment of the biological process related to ribosomes and RNA (**B**) from IP-LC-MS/MS of HTT/mHTT IP with MAB2166 or control IgG from human fibroblasts, control (17Q) and HD (69Q-het) (STRING v11.0 /IPA analysis).

Fig. S8

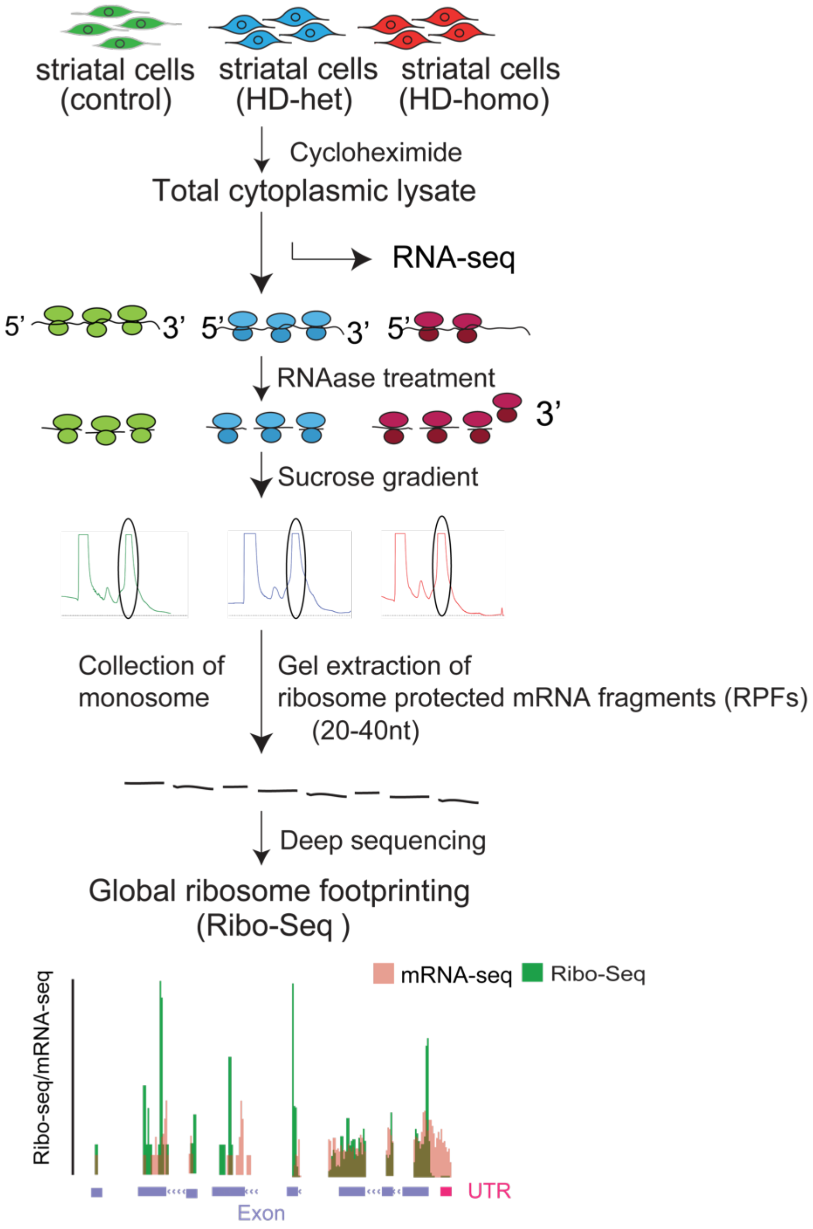

Fig. S8. **Flow Chart.** A schematic diagram showing the experimental design for performing the ribosome profiling (Ribo-Seq) and mRNA sequencing (RNA-Seq) in indicated mouse striatal cells.

Fig. S9

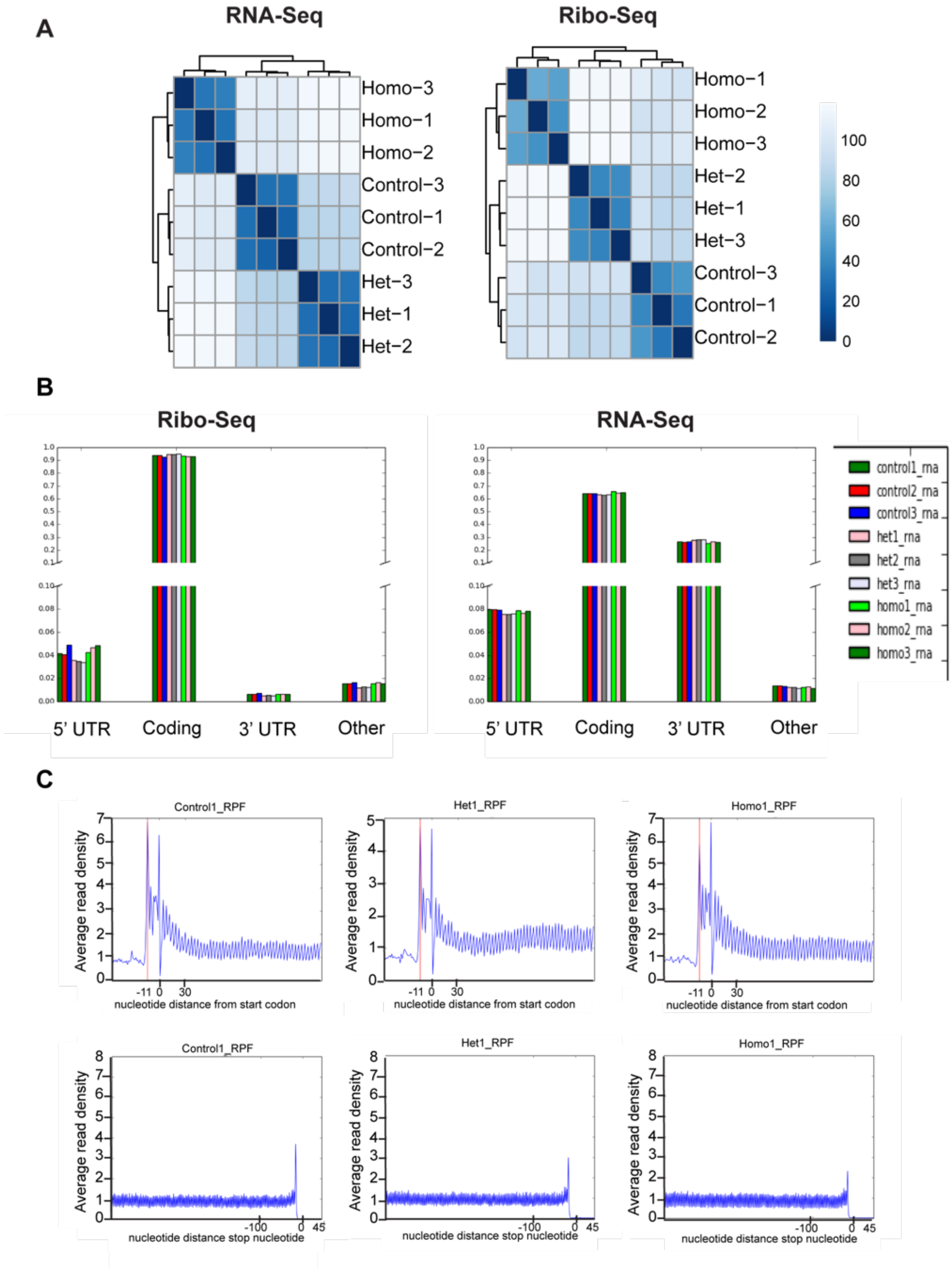

Fig. S9. Quality assessment of ribosome profiling. (**A**) Euclidian distance analyses between the biological replicates for RNA-Seq and Ribo-Seq from control, HD-het and HD-homo cells. (**B**) Ribo-Seq and RNA-Seq showing most reads are in coding region. (**C**) Ribo-Seq showing ribosome occupancy at the start and stop codon.

Fig. S10

A

B

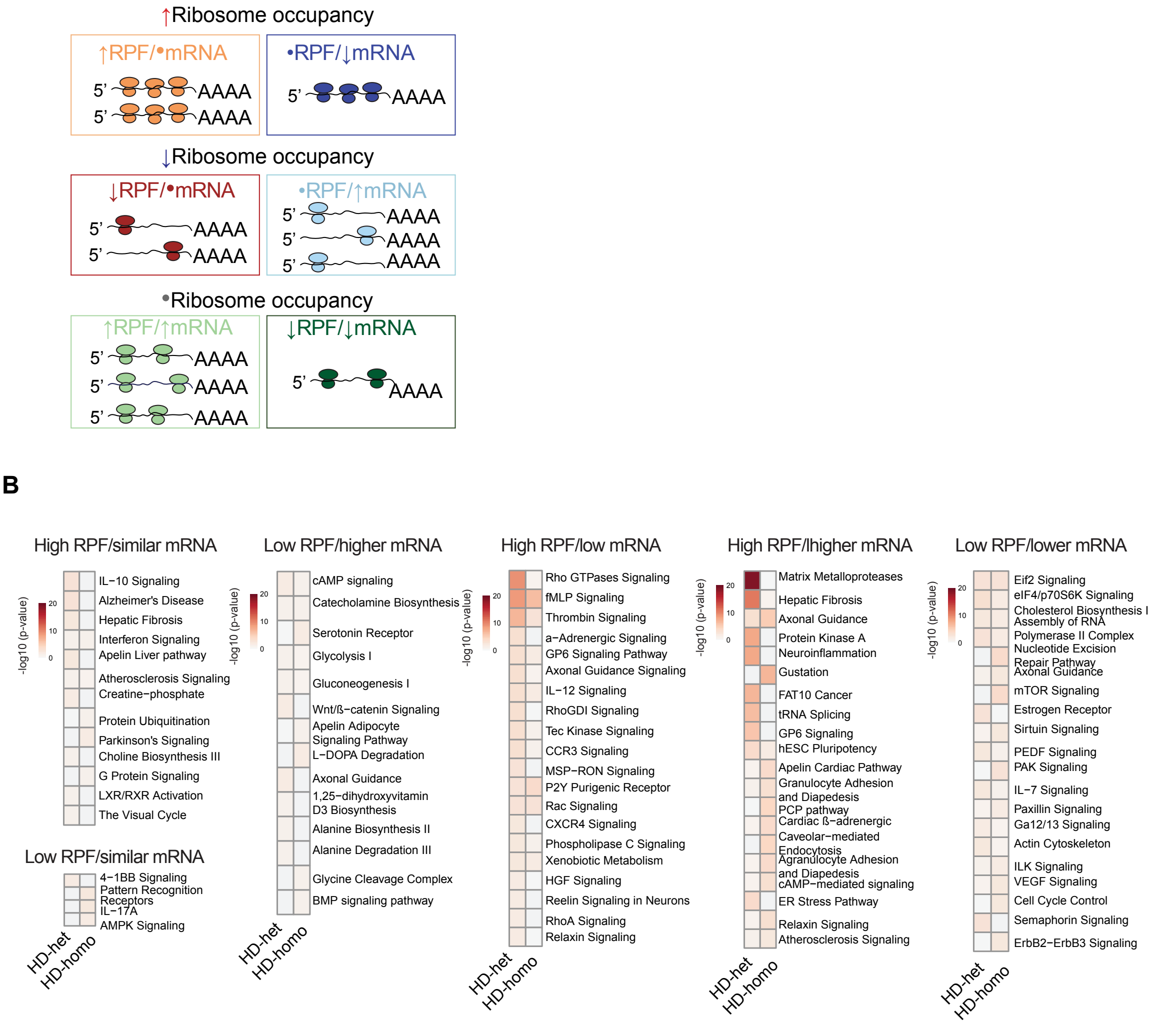

Fig. S10. (**A**) **Anota2Seq software application.** Diagram shows various types of changes in mRNA-Seq that were used to interpret Ribo-Seq data about ribosome occupancy. RPF; ribosome protected fragments. ↑: increased, ↓: decreased, and •: no change. (**B**) **IPA analysis**. Heatmap plots showing top signaling pathways dysregulated in HD-het and HD-homo cells within each group of Ribo-Seq/mRNA-Seq analyzed by ingenuity pathway analysis (IPA).

Fig. S11

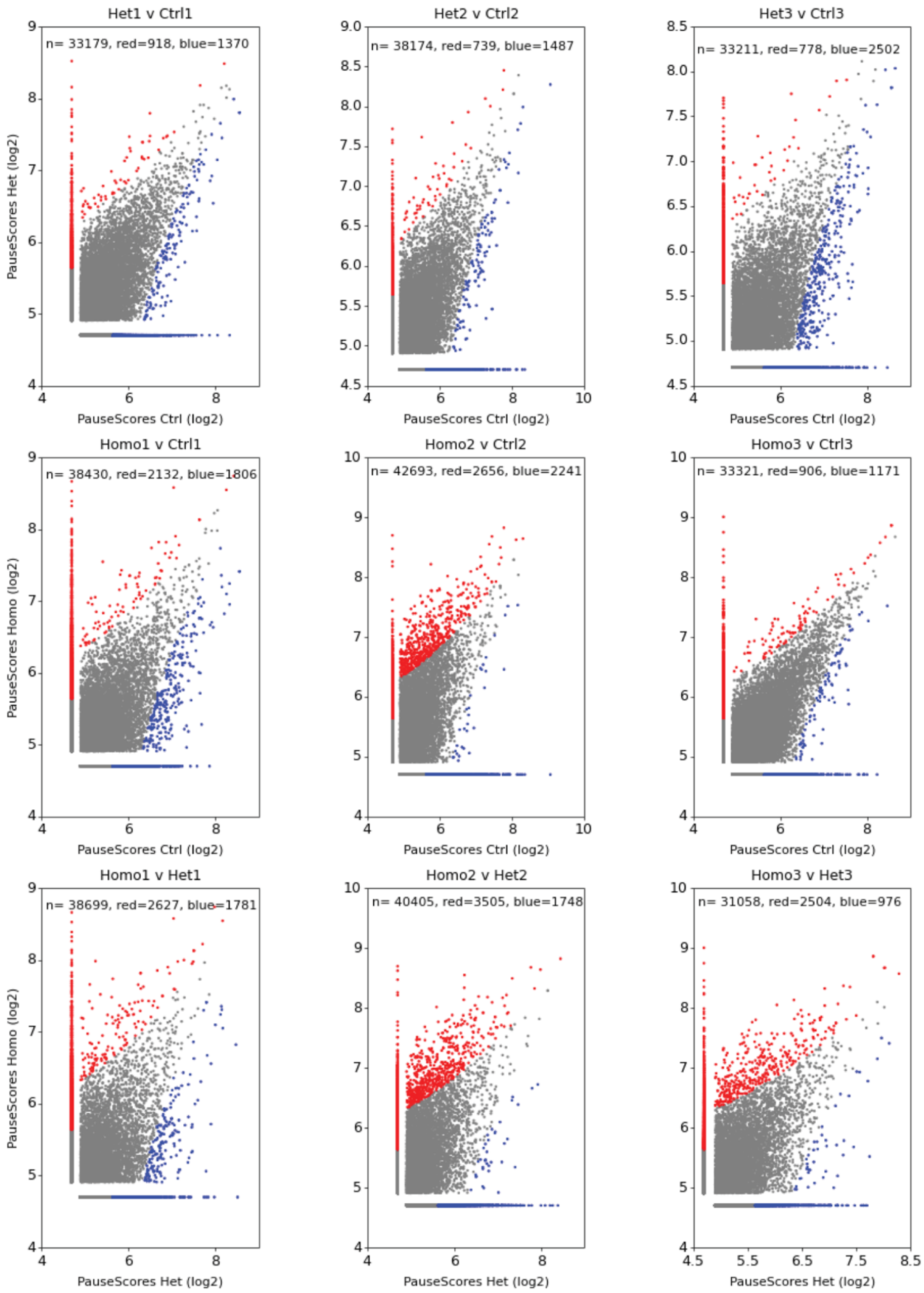
Het1 v Ctrl1
n= 33179, red=918, blue=1370
Het2 v Ctrl2
n= 38174, red=739, blue=1487
Het3 v Ctrl3
n= 33211, red=778, blue=2502
Homo1 v Ctrl1
n= 38430, red=2132, blue=1806
Homo2 v Ctrl2
n= 42693, red=2656, blue=2241
Homo3 v Ctrl3
n= 33321, red=906, blue=1171
Homo1 v Het1
n= 38699, red=2627, blue=1781
Homo2 v Het2
n= 40405, red=3505, blue=1748
Homo3 v Het3
n= 31058, red=2504, blue=976
PauseScores Het (log2)
PauseScores Homo (log2)
PauseScores Ctrl (log2)
PauseScores Het (log2)

Fig. S11. **Ribosome pause analysis.** Pause difference between the control and HD replicates show single codon pause score (fold change) of ≥ 30 for the reads of length 26-32 nucleotides with the base pair coverage requirement of 20% within a window of 1000bp. When a pause is detected in e.g., in Het1 but not in Ctrl1, it is represented in the grey/red bar along the vertical axis. The vertical grey/blue bar represents those cases where the pause is detected in Ctrl1 but not in Het1. The region between these bars represents pauses that were detected in both het1 and Ctrl1. The red cases represent pauses in Het1 that have a pause score (fold change) of >=30 compared to the same location in Ctrl1. Likewise, the blue cases represent pauses in Ctrl1 that have a pause score (fold change) of >=30 compared the same location in Het1.

Fig. S12

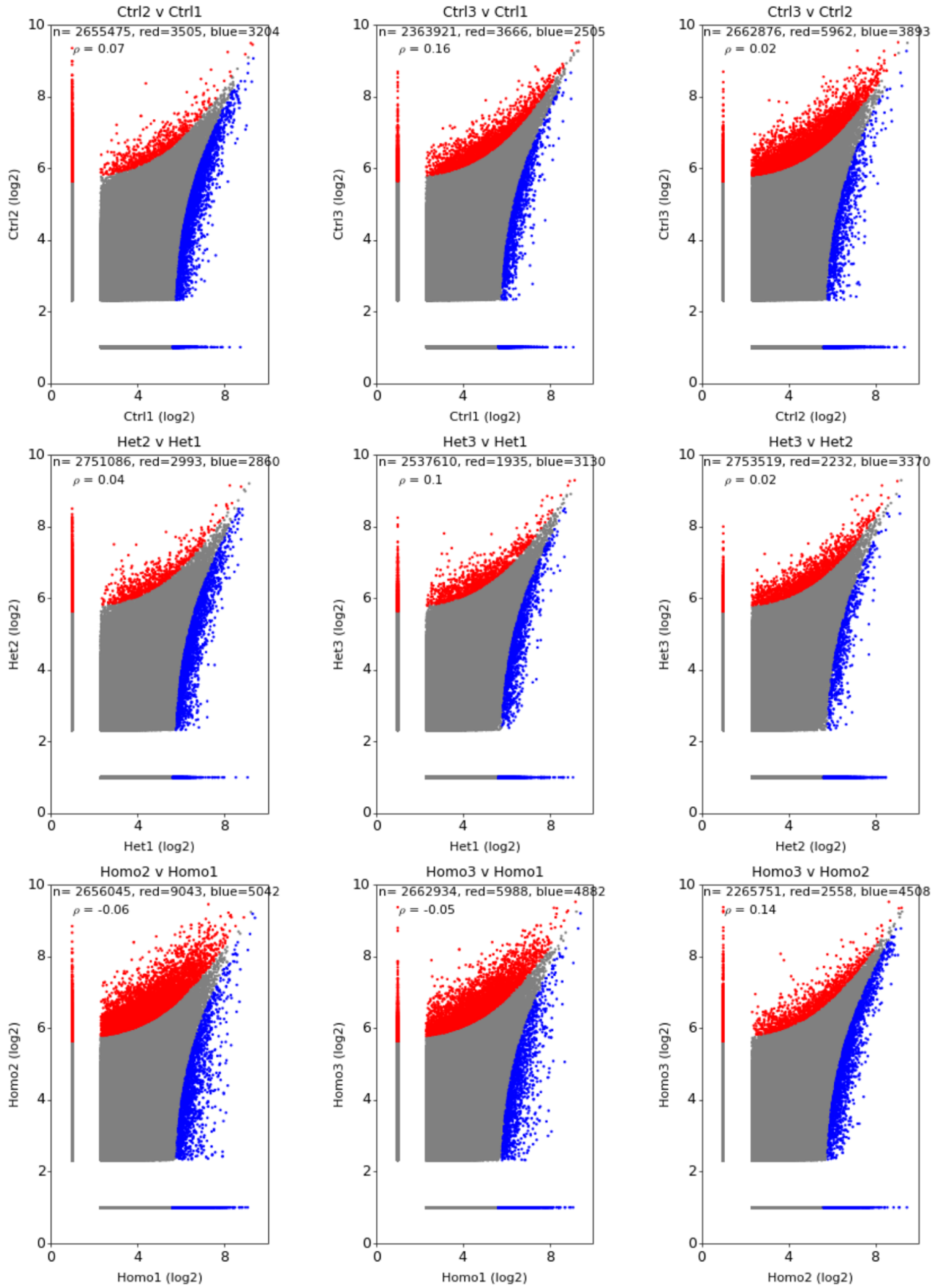
Ctrl2 v Ctrl1
n= 2655475, red=3505, blue=3204
ρ = 0.07
Ctrl2 (log2)
Ctrl1 (log2)
Ctrl3 v Ctrl1
n= 2363921, red=3666, blue=2505
ρ = 0.16
Ctrl3 (log2)
Ctrl1 (log2)
Ctrl3 v Ctrl2
n= 2662876, red=5962, blue=3893
ρ = 0.02
Ctrl3 (log2)
Ctrl2 (log2)
Het2 v Het1
n= 2751086, red=2993, blue=2860
ρ = 0.04
Het2 (log2)
Het1 (log2)
Het3 v Het1
n= 2537610, red=1935, blue=3130
ρ = 0.1
Het3 (log2)
Het1 (log2)
Het3 v Het2
n= 2753519, red=2232, blue=3370
ρ = 0.02
Het3 (log2)
Het2 (log2)
Homo2 v Homo1
n= 2656045, red=9043, blue=5042
ρ = -0.06
Homo2 (log2)
Homo1 (log2)
Homo3 v Homo1
n= 2662934, red=5988, blue=4882
ρ = -0.05
Homo3 (log2)
Homo1 (log2)
Homo3 v Homo2
n= 2265751, red=2558, blue=4508
ρ = 0.14
Homo3 (log2)
Homo2 (log2)

Fig. S12. **Ribosome pause analysis.** Pause difference within the control and HD replicates show single codon pause score (fold change) of ≥ 5 for the reads of length 26-32 nucleotides with the base pair coverage requirement of 20% within a window of 1000bp. When a pause is detected in e.g., in Ctrl1 but not in Ctrl2, it is represented in the grey/blue bar along the horizontal axis. The vertical grey/red bar represents those cases where the pause is detected in Ctrl2 but not in Ctrl1. The region between these bars represents pauses that were detected in both Ctrl2 and Ctrl1. The red cases represent pauses in Ctrl2 that have a pause score (fold change) of >=50 compared to the same location in Ctrl1. Likewise, the blue cases represent pauses in Ctrl1 that have a pause score (fold change) of >=50 compared the same location in Ctrl2. Likewise, for the remaining plots, the pause score difference was found within the control replicates and the HD replicates. The Spearman correlation for each replicate comparison within replicate groups is provided.

Supplementary Table 1

List of the IP samples from human fibroblasts for LC-MS/MS
N = 1/group

Control IgG	HTT IgG
H1- unaffected (healthy) fibroblast: Krebs	H5- unaffected (healthy) fibroblast: Krebs
H2- unaffected (healthy) fibroblasts: Krebs + Leucine	H6- unaffected (healthy) fibroblasts: Krebs + Leucine
H3- HD-het (69Q/+) fibroblasts: Krebs	H7- HD-het (69Q/+) fibroblasts: Krebs
H4- HD-het (69Q/+) fibroblasts: Krebs + Leucine	H8- HD-het (69Q/+) fibroblasts: Krebs + Leucine

Supplementary Table 2

List of human brain tissues

The subject's code (HSB#), neuropathology diagnosis (Huntington's disease grade (HD grade 1-4 or normal), age, gender, and postmortem interval (PMI) for the frozen human brain tissue (caudate nucleus).

HSB #	Neuropathology diagnosis	Age (years)	Gender	PMI (hrs)
3358	HD Grade 1	45	F	8.8
2706	HD Grade 1	43	M	17
3744	HD Grade 1	55	M	19
2858	HD Grade 2	50	M	12
3432	HD Grade 2	51	F	9.4
3635	HD Grade 2	60	F	17.5
3872	HD Grade 2	49	M	19.3
4072	HD Grade 2	50	F	16.3
4344	HD Grade 3	79	F	16
2869	HD Grade 3	67	M	15
2972	HD Grade 3	62	M	22
4518	HD Grade 3	49	M	12.7
5078	HD Grade 4	57	F	19.8
2903	HD Grade 4	58	M	7
4615	Normal	49	M	15
4823	Normal	35	F	9.3
5293	Normal	41	F	11
4340	Normal	47	M	12.5
4135	Normal	57	M	12.6

Supplementary Table 3
List of primers for real-time PCR

mGapdh_Forward	AGGTCGGTGTGAACGGATTTG
mGapdh_Reverse	TGTAGACCATGTAGTTGAGGTCA
mFMR1_Forward	CCAATGGCGCTTTCTACAAG
mFMR1_Reverse	TCTGTCTCTCTGGTTGCCAGT
mRps27_Forward	ACGACCTCCCTACGAGAACA
mRps27_Reverse	ATAGCATCCTGGGCATTTCA
hFMR1_Forward	GCAGCATGTGATGCAACTTACA
hFMR1_Reverse	CGCCTCTTTGGCACACATT
hGapdh_Forward	GGAGCGAGATCCCTCCAAAAT
hGapdh_Reverse	GGCTGTTGTCATACTTCTCATGG

m: mouse; h: human
