# Supplementary material for: Mutant Huntingtin stalls ribosomes and represses protein synthesis in a cellular model of Huntington disease
Source: Nat Commun. 2021 Mar 5;12:1461. doi: 10.1038/s41467-021-21637-y (PMC7935949; doi:10.1038/s41467-021-21637-y)
Supplement: Supplementary file 13 — Description of Additional Supplementary Files [file 41467_2021_21637_MOESM13_ESM.docx]

Your manuscript has been checked for clarity and against journal policies and formatting style. The issues listed below must be addressed; failure to do so will cause delays in acceptance.

For further information, please see our [formatting instructions](https://www.nature.com/documents/ncomms-formatting-instructions.pdf).

Please highlight all changes in the manuscript text file, either using the track changes feature in Microsoft Word or coloured highlighting in LaTeX.

Please include your response to these requests in the space provided and return this checklist with your final submission.

| **EDITORIAL REQUESTS:** | **AUTHOR RESPONSE:** |
| --- | --- |
| An updated editorial policy checklist must be completed and uploaded as a related manuscript file with the revised manuscript. All points on the policy checklist must be addressed; if needed, please revise your manuscript in response to these points. Please note that this form is a dynamic 'smart pdf' and must therefore be downloaded and completed in Adobe Reader, instead of opening it in a web browser. https://www.nature.com/authors/policies/Policy.pdf | An updated editorial policy checklist has been completed and uploaded as a related manuscript file with the revised manuscript. |
| An updated reporting summary must be completed and uploaded as a supplementary information file with the revised manuscript. All points on the reporting summary must be addressed; if needed, please revise your manuscript in response to these points. This checklist is published alongside your manuscript online. Please note that this form is a dynamic 'smart pdf' and must therefore be downloaded and completed in Adobe Reader, instead of opening it in a web browser. https://www.nature.com/authors/policies/ReportingSummary.pdf | An updated reporting summary has been completed and uploaded as a supplementary information file with the revised manuscript |
| Please find attached a reporting summary that includes comments on how to revise it in line with our policies and requests the addition of further information in the text. An updated reporting summary must be completed and uploaded as a supplementary information file with the revised manuscript. This checklist is published alongside your manuscript online. Please note that this form is a dynamic 'smart pdf' and must therefore be downloaded and completed in Adobe Reader, instead of opening it in a web browser. https://www.nature.com/authors/policies/ReportingSummary.pdf |  |
| Please also find below a list of comments requesting additional information in the figure legends, text, and methods section to comply with our reporting policies. |  |
| **TITLE PAGE (page 2 of our formatting instructions)** | **TITLE PAGE (page 2 of our formatting instructions)** |
| When discussing the current work in the abstract, please use the present tense. | Current work in the abstract has now been discussed as present tense |
| **LANGUAGE AND STYLE (page 6 of our formatting instructions)** | **LANGUAGE AND STYLE (page 6 of our formatting instructions)** |
| Please remove phrases such as 'new', 'novel', 'for the first time', 'unprecedented', etc., as novelty is clear from the context. Please also remove exaggerated language such as 'extremely', 'outstanding', etc. | These phrases have been removed from the text. |
| We do not allow statements based on data that are not present in the manuscript or unpublished. Please include all the data that is not shown or remove the statements referring to these data. | The statement referring to data not shown has been removed. |
| Please do not use italics, bold font, underlining or speech marks unless required for technical terms (in both the main text and the display items). | This has been checked. |
| Please use italics for gene names, and roman font for protein names. This applies to both the main text and display items. | Gene names are in italics. |
| Please make sure that mathematical terms throughout your manuscript and Supplementary Information (including in figures, figure axes, and legends) conform strictly to the following guidelines. Equations must be supplied in editable format, and not as images. Scalar variables (e.g. x, V, χ) must be typeset in italic, whereas multi-letter variables and functions (e.g. log) must be formatted in roman. Vectors (such as the wavevector k or the magnetic field vector B) must be typeset in bold without italics. | Done |
| **METHODS AND DATA (page 3 of our formatting instructions)** | **METHODS AND DATA (page 3 of our formatting instructions)** |
| Please rename the Methods section as 'Methods'. | Methods section has been renamed as ‘Methods’. |
| A complete list of all primers used, including the names and sequences, must be supplied as a Supplementary Table, which must be cited once in the Methods section. | Complete list of the primers is now supplied as Supplementary Table 2 and is cited in the Methods section. |
| Centrifugation speeds should be described in xg, not rpm. | Centrifuge speeds have now been described in x g |
| All manuscripts must include a Data Availability statement as a separate section after the Methods section but before the References. For acceptable examples, see: https://www.nature.com/documents/nr-data-availability-statements-data-citations.pdf The Data Availability statement should include:  - Accession codes with hyperlinks for deposited data   - Other unique identifiers (such as DOIs and hyperlinks for any other datasets)   - At a minimum, a statement confirming that all relevant data are available from the authors   - If applicable, a statement regarding data available with restrictions  - If a dataset has a Digital Object Identifier (DOI) as its unique identifier, we strongly encourage including this in the Reference list and citing the dataset in the Data Availability Statement ***Please note that the data must be released publicly by the time you resubmit your final manuscript; we will not be able to accept your manuscript if the data are not publicly available.*** | In the Data Availability section, we have now added:  The complete dataset from the analysis of the HTT interactors from healthy and HD fibroblasts (raw files, identification data, and data analysis files) can be obtained via ProteomeXchange with identifier PXD017115 at <http://www.proteomexchange.org/>.  The data for the Ribo-seq and RNA-seq reported in this study are openly available in Gene Expression Omnibus (GEO, https://www.ncbi.nlm.nih.gov/geo/) at accession number GSE146675.  UCSC browser information to view genome browser hub with the RNA-Seq and Ribo-Seq data: To find genes of your interests go to the Genome browser website (<http://genome.ucsc.edu>) and then click on “My Data > Track Hubs”. Then paste the link <https://de.cyverse.org/anon-files/iplant/home/rmi2lab/Hub_Collaborations/Srini/genome.txt> in the “url” field and click on “Add hub.” After the hub is loaded go to “Genomes > Mouse GRCm38/mm10”. Once you reach the actual browser window you will have to scroll down to the bottom menu. Find a section (the one of the top of the menu) named “Srini” to activate the different tracks. The remaining data are available within the article, supplementary Information, Source data file or available from the authors upon request. |
| Nature Research policies (https://go.nature.com/data-availability-AIP) strongly encourage deposition of research data in public repositories. In some cases this is mandatory, and you may have been previously advised if that was the case. If you need help depositing and curating your research data (including raw and processed data, text, video, audio and images) you should consider: - Contacting Springer Nature’s Research Data Helpdesk (https://go.nature.com/helpdesk-AIP) for advice - Finding a suitable data repository (https://go.nature.com/RD-policies-AIP) for your data - Uploading your data to Springer Nature’s Research Data Support service (https://go.nature.com/RDS-AIP) Research Data Support is an optional Springer Nature service. There are fees (https://go.nature.com/RDS-pricing-AIP) for using this service, however, if you receive funding from the Wellcome Trust or are affiliated to a Wellcome Centre you can use Research Data Support at no cost. See https://go.nature.com/wellcome-RDS-AIP for more information. If you choose to use Research Data Support, please do not submit your revised manuscript until you have been supplied with the DOI for your data by the Research Data Support team. Please also ensure that you update your Data Availability statement with this DOI and the information provided by the team. Please provide a unique identifier for the data (for example a DOI or a permanent URL) in the data availability statement, if possible. If the repository does not provide identifiers, we encourage authors to supply the search terms that will return the data. For data that have been obtained from publicly available sources, please provide a URL and the specific data product name in the data availability statement. Data with a DOI should be included in the reference list and cited where relevant. Alternatively, include the data in the Supplementary Information. For datasets for which mandatory deposition is not required and the data can only be shared on request, please explain why in your Data Availability Statement and in your response here.  Please refer to our data policies here: http://www.nature.com/authors/policies/availability.html |  |
| All accession codes must be accompanied with their hyperlinks throughout (for example, "5XRN [http://doi.org/10.2210/pdb5XRN/pdb]", "1483958 [https://doi.org/10.5517/ccdc.csd.cc1lt5m6]", "SRP109982 [https://www.ncbi.nlm.nih.gov/sra/?term=SRP109982]", "GSE101099 [https://www.ncbi.nlm.nih.gov/geo/query/acc.cgi?acc=GSE101099]" or "NQLW00000000 [https://www.ncbi.nlm.nih.gov/assembly/GCA_002312845.1/]"). | In the Data Availability section, we have now added:  The complete dataset from the analysis of the HTT interactors from healthy and HD fibroblasts (raw files, identification data, and data analysis files) can be obtained via ProteomeXchange with identifier PXD017115 at <http://www.proteomexchange.org/>.  The data for the Ribo-seq and RNA-seq reported in this study are openly available in Gene Expression Omnibus (GEO, https://www.ncbi.nlm.nih.gov/geo/) at accession number GSE146675.  UCSC browser information to view genome browser hub with the RNA-Seq and Ribo-Seq data: To find genes of your interests go to the Genome browser website (<http://genome.ucsc.edu>) and then click on “My Data > Track Hubs”. Then paste the link <https://de.cyverse.org/anon-files/iplant/home/rmi2lab/Hub_Collaborations/Srini/genome.txt> in the “url” field and click on “Add hub.” After the hub is loaded go to “Genomes > Mouse GRCm38/mm10”. Once you reach the actual browser window you will have to scroll down to the bottom menu. Find a section (the one of the top of the menu) named “Srini” to activate the different tracks. |
| A reference to the source data file should be added in the 'Data Availability' section, using the text “Source data are provided with this paper.” | In the Data Availability section, we have now added this sentence: The remaining data are available within the article, supplementary Information, Source data file or available from the authors upon request. |
| **END NOTES (pages 3 and 4 of our formatting instructions)** | **END NOTES (pages 3 and 4 of our formatting instructions)** |
| Please supply an 'Author Contributions' section after the 'Acknowledgements' section that specifies the contribution of every author. For more information, please see https://www.nature.com/nature-research/editorial-policies/authorship#author-contribution-statements | Author contribution section is there after the Acknowledgement section. |
| **DISPLAY ITEMS (pages 4 and 5 of our formatting instructions)** | **DISPLAY ITEMS (pages 4 and 5 of our formatting instructions)** |
| The use or adaptation of previously published images is strongly discouraged. If this is unavoidable, please request the necessary rights documentation to re-use such material from the relevant copyright holders and return this to us when you submit your revised manuscript. Please check whether your manuscript or Supplementary Information contain third-party images, such as figures from the literature, stock photos, clip art or commercial satellite and map data. |  |
| In particular, please indicate whether you or a co-author created figures- Fig- 9h | I (corresponding author) have created the figure 9h. |
| Any abbreviations, symbols or colours present in your figures must be defined in the associated legends. | This has been described in the model itself. |
| **SUPPLEMENTARY INFORMATION (page 5 of our formatting instructions)** | **SUPPLEMENTARY INFORMATION (page 5 of our formatting instructions)** |
| We do not edit Supplementary Information files; they will be uploaded with the published article as they are submitted with the final version of your manuscript. Any tracked changes should be removed from the file and the file should be provided as a PDF file. Supplementary Figures do not need to be provided separately. |  |
| Supplementary Information must be provided as a single separate PDF file, not within the manuscript file. | Supplementary Information is now provided as a single separate PDF file. |
| Every Supplementary Figure must be accompanied by a legend of up to 350 words, referring to all panels, and a brief title that summarises the whole figure. | Each Supplementary figure is accompanied with its legend and a brief title. |
| Large datasets exceeding an A4 page size should be supplied as Supplementary Data files to allow reuse, not Supplementary Tables.  ** External Databases S1-S9 should be Supplementary Data 1-9.** | External Databases S1-S9 is now Supplementary Data 1-10.  Raw data for the Violin plots (Fig. 7 C) showing the distribution of ribosome occupancy changes is included in the supplementary data file 3 |
| Please supply legends for each Supplementary Movie/Audio/Data file in your response here (not in the Supplementary Information file). Please label each files as Supplementary Movie/Audio/Data 1, etc. | **Description of Additional Supplementary Data**  **Supplementary Data 1: mRNASeq after RRA_HD-homo_vs_control**  Isolation of mRNAs from the slowly translating PS in HD-homo and compared it to the control cells, using a harringtonine-based ribosome run-off assay (RRA) followed by mRNA-Seq (PS-RRA-mRNA-Seq).  **Supplementary Data 2: IP-LC-MS/MS**  Overview spectrum counts  Total spectrum report  Publication report  **Supplementary Data 3**  Raw data for the Violin plots (Fig. 7C) showing the distribution of ribosome occupancy changes (calculated by number of Ribo-Seq reads divided by mRNA-Seq reads for each gene, Log2FC) in HD-homo and HD-het cells (compared to control cells).  **Supplementary Data 4**  mRNA targets in the HD-homo cells (RPF/mRNA) compared to the controls (p-value <0.05).  **Supplementary Data 5**  mRNA targets in the HD-het cells, with significantly changed (p-value <0.05) ribosome occupancy (RPF/mRNA) compared to the controls.  **Supplementary Data 6**  The HD-homo cells showed more genes with 5’ ribosome occupancy (5’ = 790; 3’ = 138).    **Supplementary Data 7**  HD-het cells showed more genes with 3’ occupancy (5’ = 74; 3’ = 1685).    **Supplementary Data 8**  mRNA targets in the HD-homo cells one or more codon-specific pauses  **Supplementary Data 9**  mRNA targets in the HD-het cells that showed one or more codon-specific pauses.  **Supplementary Data 10**  Single-codon paused transcripts (~130) were enriched in the top PS-bound mRNA list in PS-RRA-mRNA-Seq of the HD-homo cells. Some of the validated targets are highlighted. |
| Please ensure that a single Source Data file is included with your resubmission. Please combine current two source data files (a pdf file for raw gel images and an excel file) and provide a single file in a zip format.  Within the Source Data file, the relevant raw data from each figure or table (in the main manuscript and in the Supplementary Information) should be represented by a single sheet in an Excel document, or a single .txt file or other file type in a zipped folder. Uncropped blots and gel images should be pasted in and labelled with the relevant panel and identifying information such as the antibody used. An example of the Source Data file is available demonstrating the correct format: https://www.nature.com/documents/ncomms-example-source-data.xlsx The file should be labelled 'Source Data', with the title and a brief description included in your response here, and should be mentioned in all relevant figure legends using the template text below: "Source data are provided as a Source Data file." | Single source data zipped file is now provided which has the pdf file for raw gel images and an excel file. The file is labelled as 'Source Data' and in all the relevant figure legend we have now mentioned "Source data are provided as a Source Data file." |
| **PUBLICATION** | **PUBLICATION** |
| Your paper will be accompanied by a two-sentence Editor's summary, of between 250-300 characters including spaces, when it is published online. I have drafted the summary below. If you would like to make changes to this, please provide me with a suitably edited version. |  |
| Huntington disease (HD) is a neurodegenerative disorder caused by the expansion of a polyglutamine tract in the huntingtin (mHtt) protein. Here the authors suggest that mHtt promotes ribosome stalling and inhibits protein synthesis. | This summary is great. |
| As part of our efforts to communicate our content to a wider audience, we endeavour to highlight papers published in Nature Communications on the journal’s Twitter account (https://twitter.com/NatureComms). If you would like us to mention authors, institutions or lab groups in these tweets, please provide the relevant twitter handles. | **@scrippsresearch**  **@ScrippsFlorida** |

|  | **EDITORIAL REQUESTS:** | **AUTHOR RESPONSE:** |
| --- | --- | --- |
| **1.** | **Data presentation:** Please ensure that data presented in a plot, chart or other visual representation format shows data distribution clearly (e.g. dot plots, box-and-whisker plots). When using bar charts, please overlay the corresponding data points (as dot plots) whenever possible and always for n ≤ 10. (Please see the following editorial for the rationale behind this request and an example <https://www.nature.com/articles/s41551-017-0079>). | |
|  | **Panels requiring revision:**  Please note that data presentation has to be revised to comply with our policy in figure 4c. | Data presentation is now changed for Fig. 4c. |
| **2.** | **Statistics**:Wherever statistics have been derived (e.g. error bars, box plots, statistical significance) the legend needs to provide and define the n number (i.e. the sample size used to derive statistics) as a precise value (not a range), using the wording “n=X biologically independent samples/animals/cells/independent experiments/n= X cells examined over Y independent experiments” etc. as applicable. | |
|  | **Legends requiring revision:**   1. Please note that this information is missing in the legends of figures 4c, 6c, 7c and 9g. 2. Please provide a precise value of ‘n’ in the legends of figures 1b, 1f, 2h-i, 2k and 5b-c. | 1. the information about n number is now added in the legend of figures 4c, 6c, 7c and 9g.  2. Precise value of ‘n’ in the legends of figures 1b, 1f, 2h-i, 2k and 5b-c has now been added. |
| **3.** | Please note that statistics such as error bars significance and p values cannot be derived from n<3 and must be removed from all such cases. | |
|  | We strongly discourage deriving statistics from technical replicates, unless there is a clear scientific justification for why providing this information is important. Conflating technical and biological variability, e.g., by pooling technically replicates samples across independent experiments is strongly discouraged. (For examples of expected description of statistics in figure legends, please see the following <https://www.nature.com/articles/s41467-019-11636-5> or <https://www.nature.com/articles/s41467-019-11510-4>). | |
|  | All error bars need to be defined in the legends (e.g. SD, SEM) together with a measure of centre (e.g. mean, median). For example, the legends should state something along the lines of “Data are presented as mean values +/- SEM” as appropriate.  All box plots need to be defined in the legends in terms of minima, maxima, centre, bounds of box and whiskers and percentile. | |
|  | **Legends requiring revision:**   1. Please note that the error bars need to be defined in the legends of figures 4c and 9g. 2. Please note that the box plots need to be defined in terms of minima, maxima, centre, bounds of box and whiskers and percentile in the legends of figures 6c and 7c. | 1. Error bars are now defined in the legends of figures 4c and 9g.  2. Box plots are now defined in the legends of figures 6c and 7c. |
| **4.** | The figure legends must indicate the statistical test used. Where appropriate, please indicate in the figure legends whether the statistical tests were one-sided or two-sided and whether adjustments were made for multiple comparisons.  For null hypothesis testing, please indicate the test statistic (e.g. F, t, r) with confidence intervals, effect sizes, degrees of freedom and P values noted.  Please provide the test results (e.g. P values) as exact values whenever possible and with confidence intervals noted. | |
|  | **Legends requiring revision:**   1. Please indicate the statistical test used for data analysis and where appropriate, please specify whether it was one-sided or two-sided and whether adjustments were made for multiple comparisons, in the legends of figures 4b, 4c, 7d and 8d; supplementary figures 7b and 10b. 2. Please note that the information on whether the statistical test used was one-sided or two-sided, where appropriate, is missing in the legends of figures 2e-f, 2h-i, 2k, 3b and 4d-e; supplementary figures 2b, 3b and 4b. 3. Please note that the exact p value should be provided in the source data file. (figures 1b, 1d, 1f, 1h, 2b-c, 2f, 2i, 2k, 3b, 3c, 3d, 4d-e, 5b-c, 5e and 9g; supplementary figures 2b, 3b and 4b.) 4. Please indicate what ‘*’ represents; if this represents p values, please indicate the statistical test used and where appropriate, specify whether it was one-sided or two-sided and whether adjustments were made for multiple comparisons and the exact p value in the source data file. 5. For figure 9g, we observed that ** representation has been provided in the panel but not in the legend; and *** representation has been provided in the legend but not in the panel. Please rectify the representation in the legend or panel appropriately and provide the exact p value in the source data file. | 1. Statistical test details are now mentioned in legends for figures 4b, 4c, 7d and 8d; supplementary figures 7b and 10b.  2. Two-tailed Student’s t test information has been added in legends of figures 2e-f, 2h-i, 2k, 3b and 4d-e; supplementary figures 2b, 3b and 4b.  3. Exact p value (figures 1b, 1d, 1f, 1h, 2b-c, 2f, 2i, 2k, 3b, 3c, 3d, 4d-e, 5b-c, 5e and 9g; supplementary figures 2b, 3b and 4b.) is now provided in the source data file  4. * or # represents p value and has been added in the source data file  5. representation in the legend for figure 9g has been rectified and exact p value is in source data file. |
| **5.** | **Reproducibility:** Please state in the legends how many times each experiment was repeated independently with similar results. This is needed for all experiments, but is particularly important wherever results from representative experiments (such as micrographs) are shown. If space in the legends is limiting, this information can be included in a section titled “Statistics and Reproducibility” in the methods section. | |
|  | **Legends requiring revision:**  Please note that this information is missing in the legends of figures 3a, 6a, 6b and 6d-e; supplementary figure 5. | legends of figures 3a, 6a, 6b and 6d-e; supplementary figure 5 has the n value. |
| **6.** | **Data availability:**This journal strongly supports public availability of data and custom code associated with the paper in a persistent repository where they can be freely and enduringly accessed or as a supplementary data file when no appropriate repository is available. If data and code can only be shared on request, please explain why in your data Availability Statement, and also in the correspondence with your editor. For more information, please refer to <https://www.nature.com/nature-research/editorial-policies/reporting-standards#availability-of-data> | |
|  | Please ensure that datasets deposited in public repositories are now publicly accessible, and that accession codes or DOI are provided in the "Data Availability" section. As long as these datasets are not public, we cannot proceed with the acceptance of your paper. For data that have been obtained from publicly available sources, please provide a URL and the specific data product name in the data availability statement. Data with a DOI should be further cited in the methods reference section. | In the Data Availability section, we have now added: The data for the Ribo-seq and RNA-seq reported in this study are openly available in Gene Expression Omnibus (GEO, https://www.ncbi.nlm.nih.gov/geo/) at accession number GSE146675.  UCSC browser information to view genome browser hub with the RNA-Seq and Ribo-Seq data: To find genes of your interests go to the Genome browser website (<http://genome.ucsc.edu>) and then click on “My Data > Track Hubs”. Then paste the link <https://de.cyverse.org/anon-files/iplant/home/rmi2lab/Hub_Collaborations/Srini/genome.txt> in the “url” field and click on “Add hub.” After the hub is loaded go to “Genomes > Mouse GRCm38/mm10”. Once you reach the actual browser window you will have to scroll down to the bottom menu. Find a section (the one of the top of the menu) named “Srini” to activate the different tracks. |
| **7.** | **Gels and Blots:** Quantitative comparisons between samples on different gels/blots are discouraged; if this is unavoidable, the figure legend must state that the samples derive from the same experiment and that gels/blots were processed in parallel.  Vertically sliced images that juxtapose lanes that were non-adjacent in the gel must have a clear separation or a black line delineating the boundary between the gels. Loading controls (e.g. GAPDH, actin) must be run on the same blot.  Sample processing controls run on different gels must be identified as such in the figure legends, and distinctly from loading controls.  All blots and gels must be accompanied by the locations of molecular weight/size markers. Blots should be cropped such that at least one marker position is present.  Please also supply uncropped and unprocessed scans of the most important blots in the Source Data file or as a supplementary figure in the Supplementary Information. This should be cited once in the Methods section. For an example of presentation of full scan blots, see the Source Data file of <https://www.nature.com/articles/s41467-020-16984-1#Sec35> and for more information, please refer to <https://www.nature.com/nature-research/editorial-policies/image-integrity> | |
|  | **Panels requiring revision:**  Please note that molecular weight markers are missing for figures 1c, 1g, 2a, 4d-e, 5a, 5d, 6a, 6d-e and 9g; supplementary figures 2a and 3a. | Molecular weight markers have been added for figures 1c, 1g, 2a, 4d-e, 5a, 5d, 6a, 6d-e and 9g; supplementary figures 2a and 3a. |
| **8.** | **Micrographs:** Please ensure that all micrographs include a scale bar and this scale bar is defined on the panels or in the figure legends.  All micrographs include scale bar that is defined. | |
