# Supplementary material for: Mutant Huntingtin stalls ribosomes and represses protein synthesis in a cellular model of Huntington disease
Source: Nat Commun. 2021 Mar 5;12:1461. doi: 10.1038/s41467-021-21637-y (PMC7935949; doi:10.1038/s41467-021-21637-y)
Supplement: Supplementary file 14 — Reporting Summary [file 41467_2021_21637_MOESM14_ESM.pdf]

nature research

Corresponding author(s): Srinivasa Subramaniam

Last updated by author(s): Jan 22, 2021

Reporting Summary

Nature Research wishes to improve the reproducibility of the work that we publish. This form provides structure for consistency and transparency in reporting. For further information on Nature Research policies, see Authors & Referees and the Editorial Policy Checklist.

Statistics

For all statistical analyses, confirm that the following items are present in the figure legend, table legend, main text, or Methods section.

n/a	Confirmed	
☐	☒	The exact sample size (*n*) for each experimental group/condition, given as a discrete number and unit of measurement
☐	☒	A statement on whether measurements were taken from distinct samples or whether the same sample was measured repeatedly
☐	☒	The statistical test(s) used AND whether they are one- or two-sided<br>*Only common tests should be described solely by name; describe more complex techniques in the Methods section.*
☒	☐	A description of all covariates tested
☐	☒	A description of any assumptions or corrections, such as tests of normality and adjustment for multiple comparisons
☐	☒	A full description of the statistical parameters including central tendency (e.g. means) or other basic estimates (e.g. regression coefficient) AND variation (e.g. standard deviation) or associated estimates of uncertainty (e.g. confidence intervals)
☐	☒	For null hypothesis testing, the test statistic (e.g. *F*, *t*, *r*) with confidence intervals, effect sizes, degrees of freedom and *P* value noted<br>*Give P values as exact values whenever suitable.*
☒	☐	For Bayesian analysis, information on the choice of priors and Markov chain Monte Carlo settings
☒	☐	For hierarchical and complex designs, identification of the appropriate level for tests and full reporting of outcomes
☒	☐	Estimates of effect sizes (e.g. Cohen's *d*, Pearson's *r*), indicating how they were calculated

Our web collection on statistics for biologists contains articles on many of the points above.

Software and code

Policy information about availability of computer code

Data collection	For Riboseq reads, 3' adapters were trimmed using Cutadapt v1.18 and reads were mapped using Bowtie (versoin 1.1.2). Fastq files were checked for quality control with FastQC v0.11.8. PausePred software (Andreev et al., 2015b; Kumari et al., 2018) was utilized for ribosome density measurements. For RNA-seq, reads were mapped using RSEM v1.3.0 and differential gene expression analysis was done using DESeq2 v1.22.2. Cleaned bam files were converted to bigWig files with Bedtools v2.27.0 for visualisation using the UCSC Genome Browser. For the euclidian distance analyses, gene expression was quantified with RSEM v1.3.0 and comparison plots were generated in R using DESeq2 v1.22.2 and ggplot2 v3.3.0 packages.
Data analysis	The colocalization analysis itself was performed using the ImageJ plugin JACoP, utilizing both the pixel-based Manders coefficient analysis and the object-based methods. Anato2Seq software v1.5.2 was used for Ribosome occupancy analysis. The command line version of the PausePred software (Andreev et al., 2015b; Kumari et al., 2018) was used for Ribosome pause (PausePred) analysis. For the euclidian distance analyses, gene expression was quantified with RSEM v1.3.0 and comparison plots were generated in R using DESeq2 v1.22.2 and ggplot2 v3.3.0 packages. Protein identification was carried out using Sequest algorithms (Proteome Discoverer v1.4, Thermo Scientific). Scaffold (version Scaffold_4.8.8, Proteome Software Inc., Portland, OR) was used to validate MS/MS based peptide and protein identifications. GraphPad Prism 7 was used in analysis of all other data presented in the manuscript.

For manuscripts utilizing custom algorithms or software that are central to the research but not yet described in published literature, software must be made available to editors/reviewers. We strongly encourage code deposition in a community repository (e.g. GitHub). See the Nature Research guidelines for submitting code & software for further information.

Data

Policy information about availability of data

All manuscripts must include a data availability statement. This statement should provide the following information, where applicable:

- Accession codes, unique identifiers, or web links for publicly available datasets
- A list of figures that have associated raw data
- A description of any restrictions on data availability

Data Availability
The complete dataset from the analysis of the HTT interactors from healthy and HD fibroblasts (raw files, identification data, and data analysis files) can be obtained via ProteomeXchange with identifier PXD017115 at http://www.proteomexchange.org/.
The data for the Ribo-seq and RNA-seq reported in this study are openly available in Gene Expression Omnibus (GEO, https://www.ncbi.nlm.nih.gov/geo/) at accession number GSE146675.
UCSC browser information to view genome browser hub with the RNA-Seq and Ribo-Seq data: To find genes of your interests go to the Genome browser website (http://genome.ucsc.edu) and then click on "My Data > Track Hubs". Then paste the link https://de.cyverse.org/anon-files/iplant/home/rmi2lab/Hub_Collaborations/Srini/genome.txt in the "url" field and click on "Add hub." After the hub is loaded go to "Genomes > Mouse GRCm38/mm10". Once you reach the actual browser window you will have to scroll down to the bottom menu. Find a section (the one of the top of the menu) named "Srini" to activate the different tracks. The remaining data are available within the article, supplementary Information, Source data file or available from the authors upon request.

Field-specific reporting

Please select the one below that is the best fit for your research. If you are not sure, read the appropriate sections before making your selection.

☒ Life sciences ☐ Behavioural & social sciences ☐ Ecological, evolutionary & environmental sciences

For a reference copy of the document with all sections, see nature.com/documents/nr-reporting-summary-flat.pdf

Life sciences study design

All studies must disclose on these points even when the disclosure is negative.

Sample size	As shown in the figure legends, all cell culture experiments involving statistical analysis were performed at least 3 times. Sample size determination was not used as there was no in vivo experiments performed.
Data exclusions	No data were excluded from analyses.
Replication	Cell culture, biochemical and Ribo-Seq experiments were repeated minimum 3 times to ensure reproducibility and all the attempts were successful. For PS-RRA-mRNA-Seq experiments where the ribosomes are isolated for further mRNA seq experiments, the N=2 was used, which was deemed sufficient as it was built upon the Ribo-Seq experiment with higher power with N=3 per group. We also used the PS-RRA-mRNA-Seq experiment to reconfirm the increased expression of Fmrp (which was confirmed with qPCR, N= 8) or ribosomal pauses (Ribo-seq, N =3). In IP-LC-MS/MS experiment with human fibroblasts, 4 control IgG and 4 HTT IgG samples were analyzed (N = 1 per group).
Randomization	There was no particular randomization process used for cell culture or biochemical experiments. Cell lines and in vitro biochemical experiments are well-characterized and the manuscript does not involve animals or clinical investigation.
Blinding	Blinding was employed in IP-LC-MS/MS experiments with human fibroblasts. Partial blinding was employed in the Ribo-Seq experiments, where the library was prepared with blinding codes. Most other experiments used routine cell culture and biochemical tools and the risk of bias are very low.

Reporting for specific materials, systems and methods

We require information from authors about some types of materials, experimental systems and methods used in many studies. Here, indicate whether each material, system or method listed is relevant to your study. If you are not sure if a list item applies to your research, read the appropriate section before selecting a response.

Materials & experimental systems

n/a	Involved in the study	
☐	☒	Antibodies
☐	☒	Eukaryotic cell lines
☒	☐	Palaeontology
☒	☐	Animals and other organisms
☐	☒	Human research participants
☒	☐	Clinical data

Methods

n/a	Involved in the study	
☒	☐	ChIP-seq
☒	☐	Flow cytometry
☒	☐	MRI-based neuroimaging

Antibodies

Antibodies used	The following commercial antibodies were used: Huntingtin (MAB2166, 1:3000) and puromycin (MABE343, 1:10000) antibodies were obtained from, Millipore-Sigma. Anti-polyglutamine (poly-Q) antibody (P1874, 1:5000) was from Sigma. Actin (sc47778, 1:20000) and GST-horseradish peroxidase (HRP, sc138 HRP, 1:10000) antibodies were from Santa Cruz Biotechnology. RPL7 (IHC-00455, 1:10000), RPL35A (A305-106A, 1:10,000) and Caprin1 (A303-881A, 1:1000) from Bethyl Laboratories. mTOR (2972, 1:3000), FMRP (4317, 1:1500), and S6 (2217, 1:10000) and normal mouse IgG (5415, 1:2500) were from Cell Signaling Technology. Mfsd (10 11518-1-527 AP, 1:1000), Acan (13880-1-AP, 1:1000), Ppbp (13313-1-AP, 1:1000), Mgp (10734-1-AP, 1:1000), and Phf11d (10898-1-AP, 1:1000) were from Proteintech. HRP-conjugated secondary antibodies were from Jackson ImmunoResearch Inc. HRP-conjugated secondary antibodies [115-035-146 (goat anti-mouse), 1:10,000; or 111-035-144 (goat anti-rabbit), 1:10,000] were from Jackson ImmunoResearch Inc. For Immunostaining Huntingtin (1:100, MAB2166), Rpl7 (1:50; IHC-00455) were used. Secondary antibody for STED microscopy were Anti-mouse STAR 635p (1:400) and Anti-rabbit Alexa 594 (1:400) were self-coupled in house and the company that produced is Abberior, Göttingen, Germany.
Validation	The following commercial antibodies were used: Huntingtin (MAB2166, 1:3000) and puromycin (MABE343, 1:10000) antibodies were obtained from, Millipore-Sigma and validated by the manufacture for the application and species. See manufacturer website for the validation information. www.emdmillipore.com. For Immunostaining using STED, the huntingtin antibody MAB2166 was used at 1:100 as recommended.<br>Anti-polyglutamine (poly-Q) antibody (P1874, 1:5000) was from Sigma and validated by the manufacture for the application and species, see the website for more information. www.sigmaaldrich.com<br>Actin (sc47778, 1:20000) and GST-horseradish peroxidase (HRP, sc138 HRP, 1:10000) antibodies were from Santa Cruz Biotechnology and validated by the manufacture for Western and species. See the link for the details. www.scbt.com<br>RPL7 (IHC-00455, 1:10000), RPL35A (A305-106A, 1:10,000) and Caprin1 (A303-881A, 1:1000) from Bethyl Laboratories, and they have been validated by the manufacture for immunocytochemistry and Western blot. www.bethyl.com For Immunostaining by STED, the Rpl7 antibody IHC-00455 was used at 1:50<br>mTOR (2972, 1:3000), FMRP (4317, 1:1500), and S6 (2217, 1:10000) and normal mouse IgG (5415, 1:2500) were from Cell Signaling Technology, who also validated it for the Western blotting. See more information in the weblink. www.cellsignal.com<br>Antibodies Mfsd (10 11518-1-527 AP, 1:1000), Acan (13880-1-AP, 1:1000), Ppbp (13313-1-AP, 1:1000), Mgp (10734-1-AP, 1:1000), and Phf11d (10898-1-AP, 1:1000) were from Proteintech. They are validated for Western blotting by the manufacture www.ptglab.com<br>HRP-conjugated secondary antibodies were from Jackson ImmunoResearch Inc. HRP-conjugated secondary antibodies [115-035-146 (goat anti-mouse), 1:10,000; or 111-035-144 (goat anti-rabbit), 1:10,000] were from Jackson ImmunoResearch Inc. Further information about these antibody and dilution can be obtained from www.jacksonimmuno.com<br><br>Using multiple cell lines we confirmed the expected size of the protein band in western blotting. Furthermore, CRISPR/Cas9 targeting of Htt and FMRP revealed loss of the signals in western blotting with these antibodies indicating the specificity.

Eukaryotic cell lines

Policy information about cell lines

Cell line source(s)	The mouse striatal cells (STHdh), control (STHdhQ7/Q7), HD-het (STHdhQ7/Q111), and HD-homo (STHdhQ111/Q111) and normal human fibroblast cell line (GM07492) and HD patient-derived fibroblast cell lines (GM04281, wild type HTT allele/17 CAG repeats, mutant HTT allele/69 CAG repeats) were obtained from the Coriell Institute for Medical Research (Camden, New Jersey, USA)
Authentication	Cells are authenticated by the provider as well as numerous independent studies
Mycoplasma contamination	We routinely test our cells line for mycoplasma contamination. Cells used in this study are devoid of Mycoplasma as tested by PCR analysis.
Commonly misidentified lines (See ICLAC register)	Commonly misidentified lines were not used in this study.

Human research participants

Policy information about studies involving human research participants

Population characteristics	Only patient-derived brain samples were used. Frozen human brain tissue (Caudate nucleus) samples of grade 1 HD-affected patient, grade 2 HD-affected patient, grade 3 HD-affected patient, grade 4 HD-affected patient and normal donor controls were obtained from the the NIH Neurobiobank (Human Brain and Spinal Fluid Resource Center, VA West Los Angeles Healthcare Center, 11301 Wilshire Blvd. Los Angeles, CA 90073 which is supported in part by National Institutes of Health (HHSN-271-201300029C) and the US Department of Veterans Affairs).
Recruitment	All frozen tissue samples were obtained retrospectively. No patients were recruited for this study.
Ethics oversight	Human tissue obtained from the NIH NeuroBioBank (Human Brain and Spinal Fluid Resource Center) was with informed consent from the donors. This was overseen by institutional review board PCC#: 2015–060672, VA Project #: 0002 and were analyzed under ethical and safety guidelines approved by the Scripps Research Institute and its Institutional Review Board.

Note that full information on the approval of the study protocol must also be provided in the manuscript.
