# Supplementary figures and images for: Mutant Huntingtin stalls ribosomes and represses protein synthesis in a cellular model of Huntington disease
Source: Nat Commun. 2021 Mar 5;12:1461. doi: 10.1038/s41467-021-21637-y (PMC7935949; doi:10.1038/s41467-021-21637-y)

**Raw Data files:**  
**Fig. 1C**

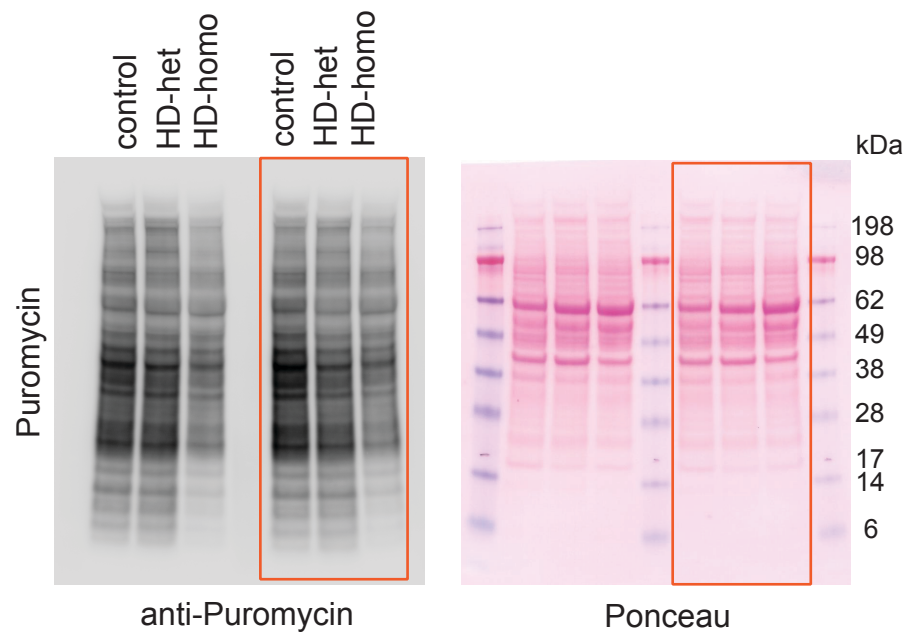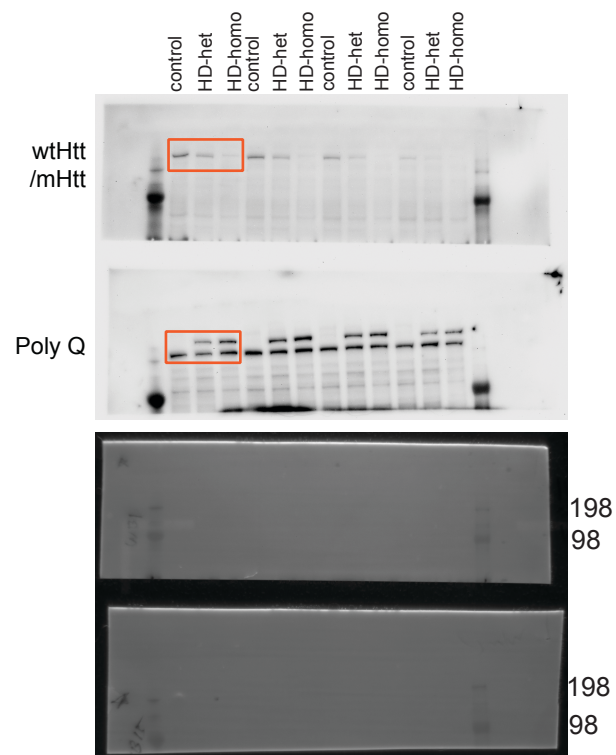

**Fig. 1G**

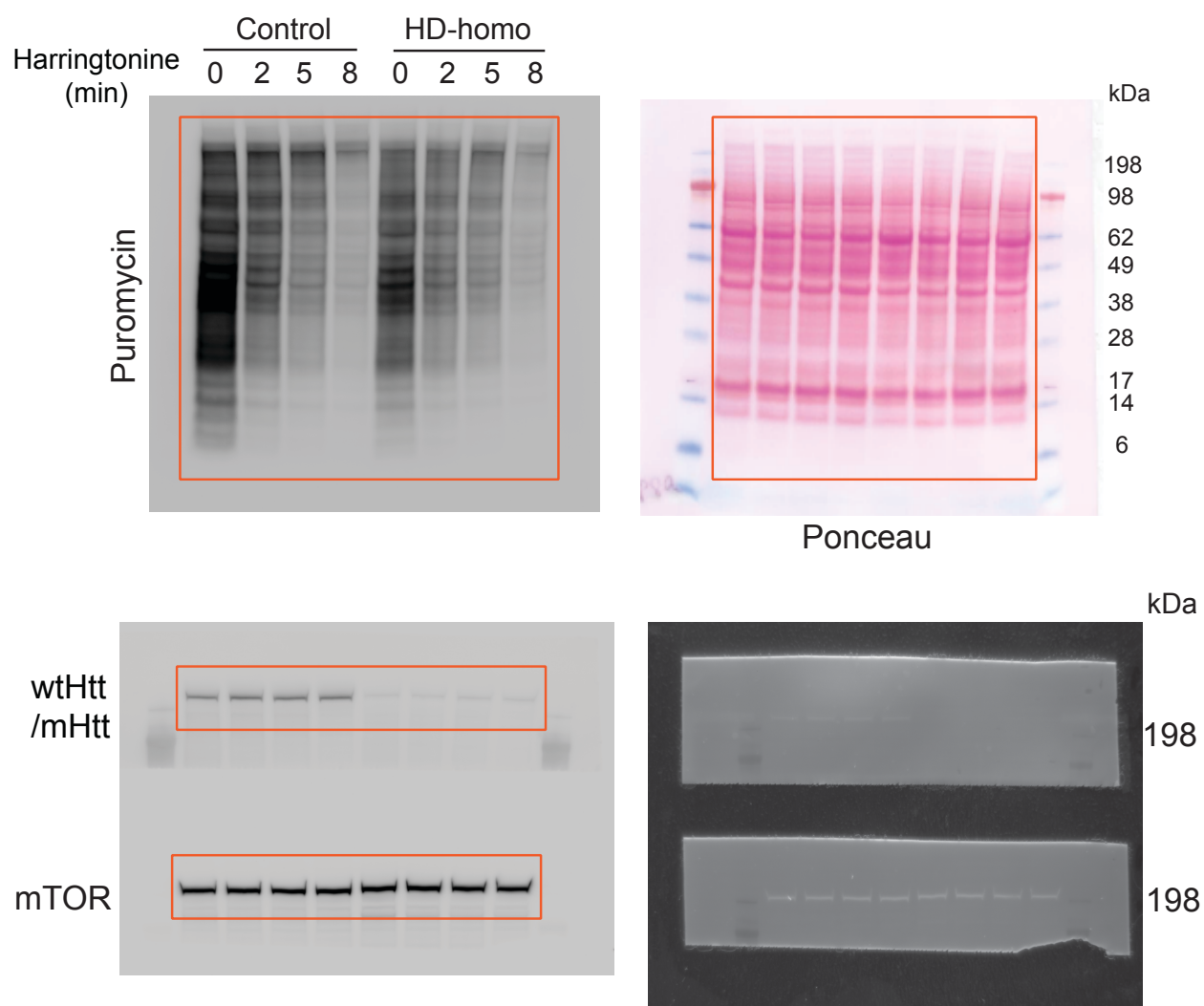

Fig. 2A

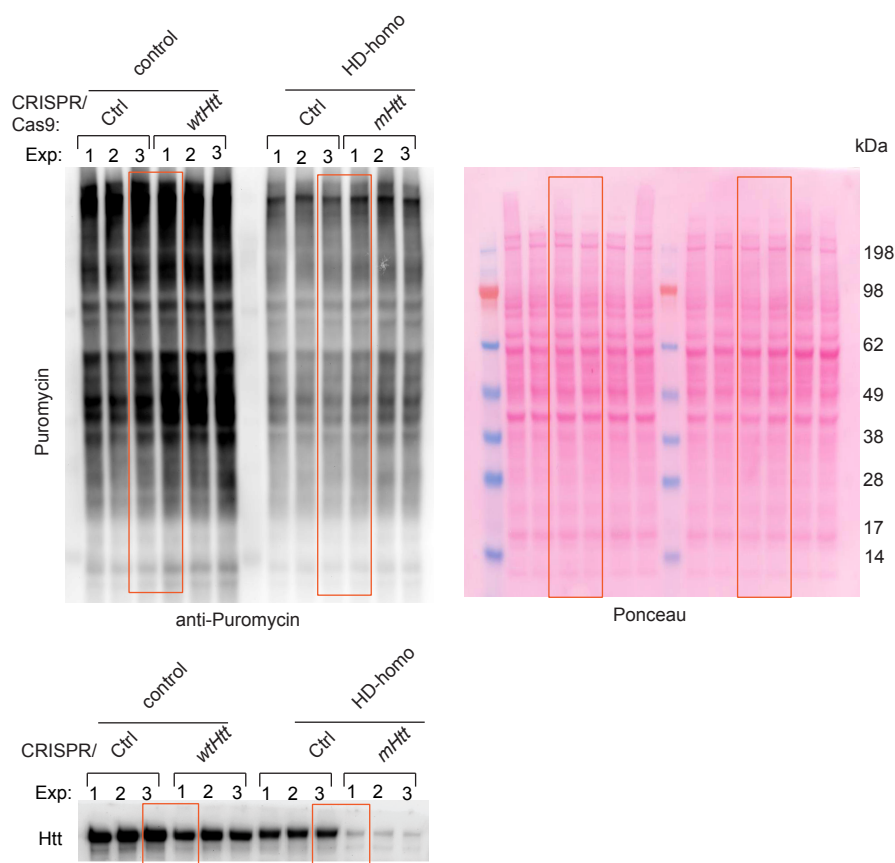

**Fig. 4D**

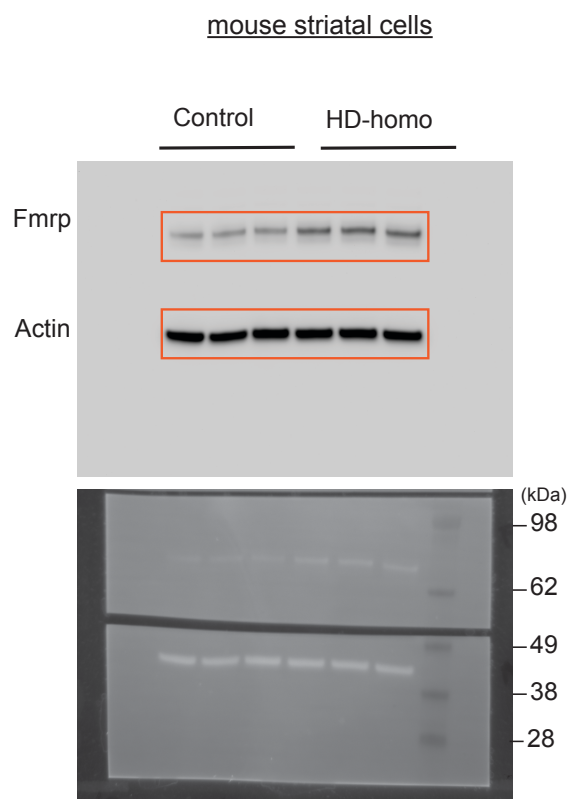

**Fig. 4E**

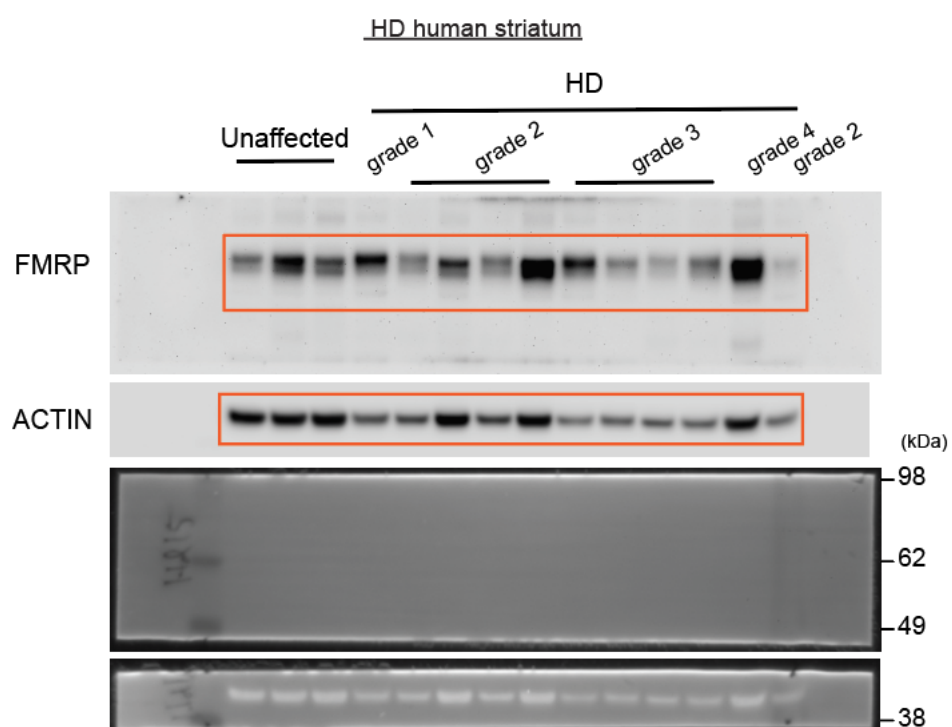

**Fig 5A**

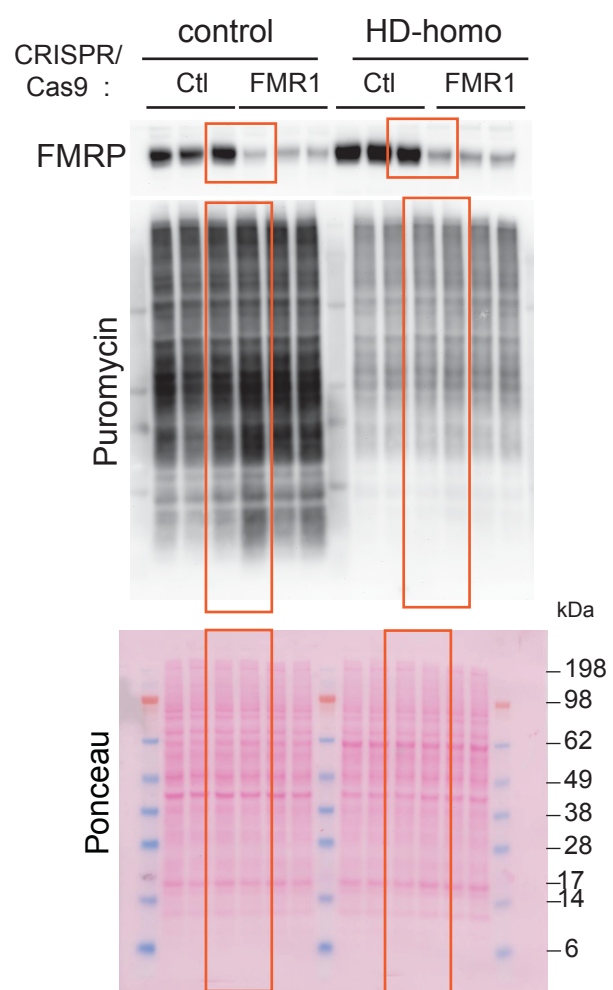

**Fig. 5E**

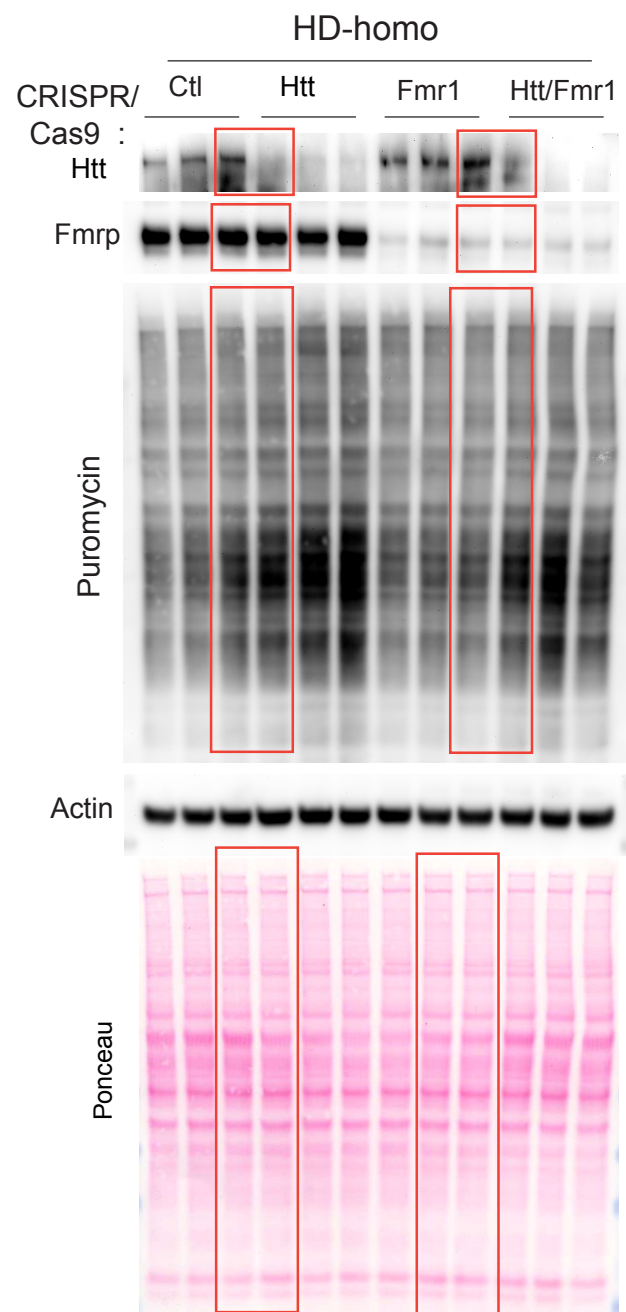

**Fig. 6A**

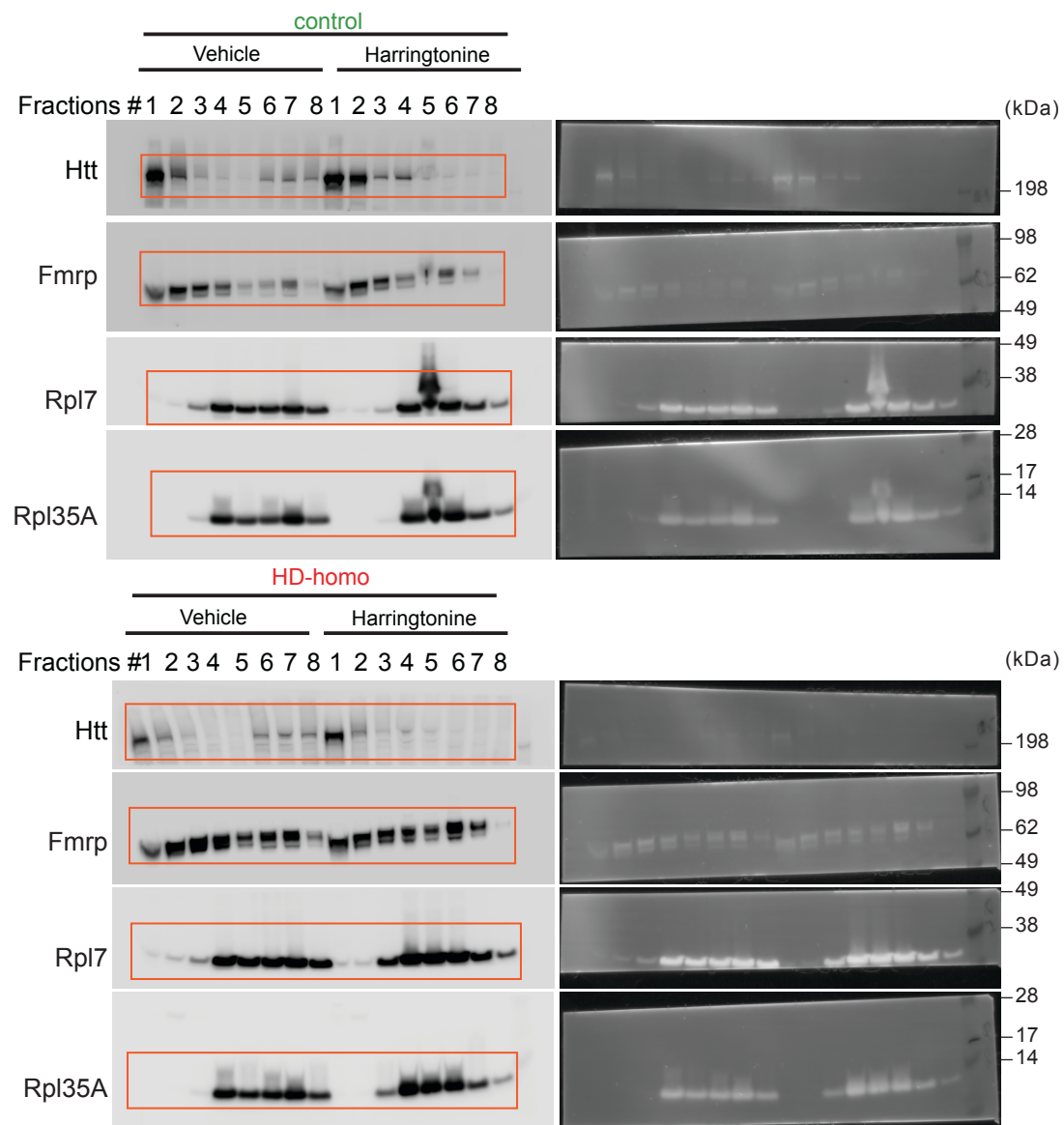

**Fig. 6D**

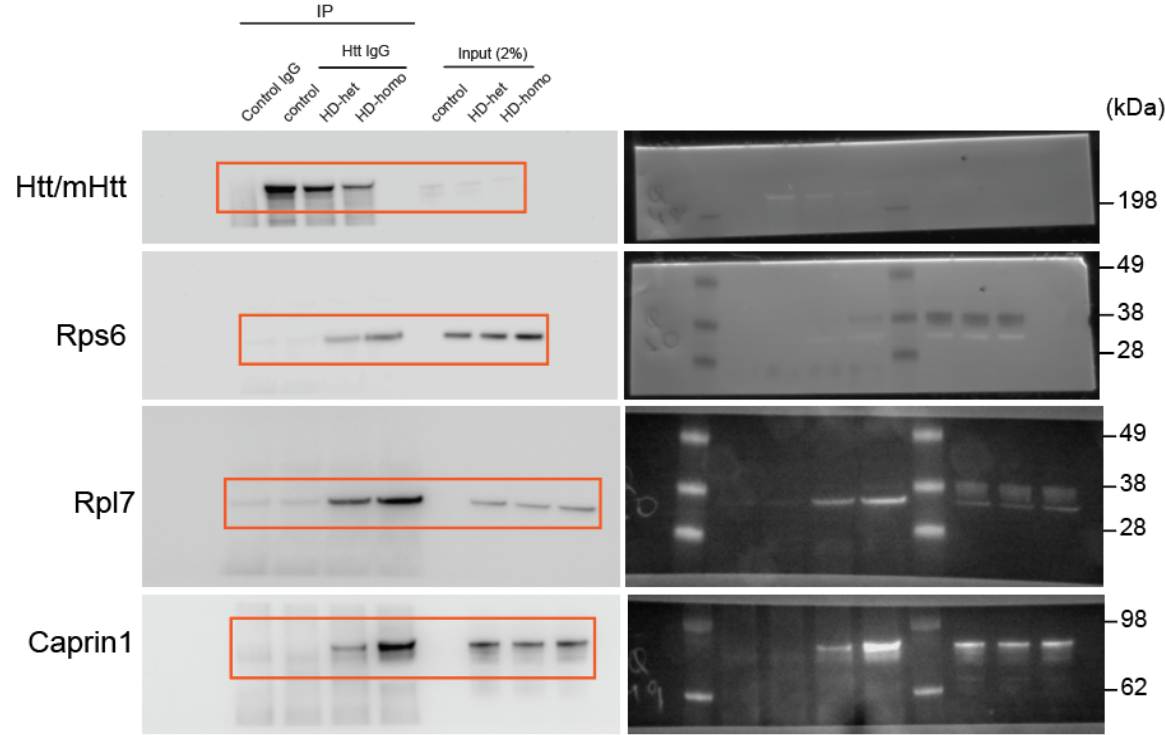

**Fig. 6E**

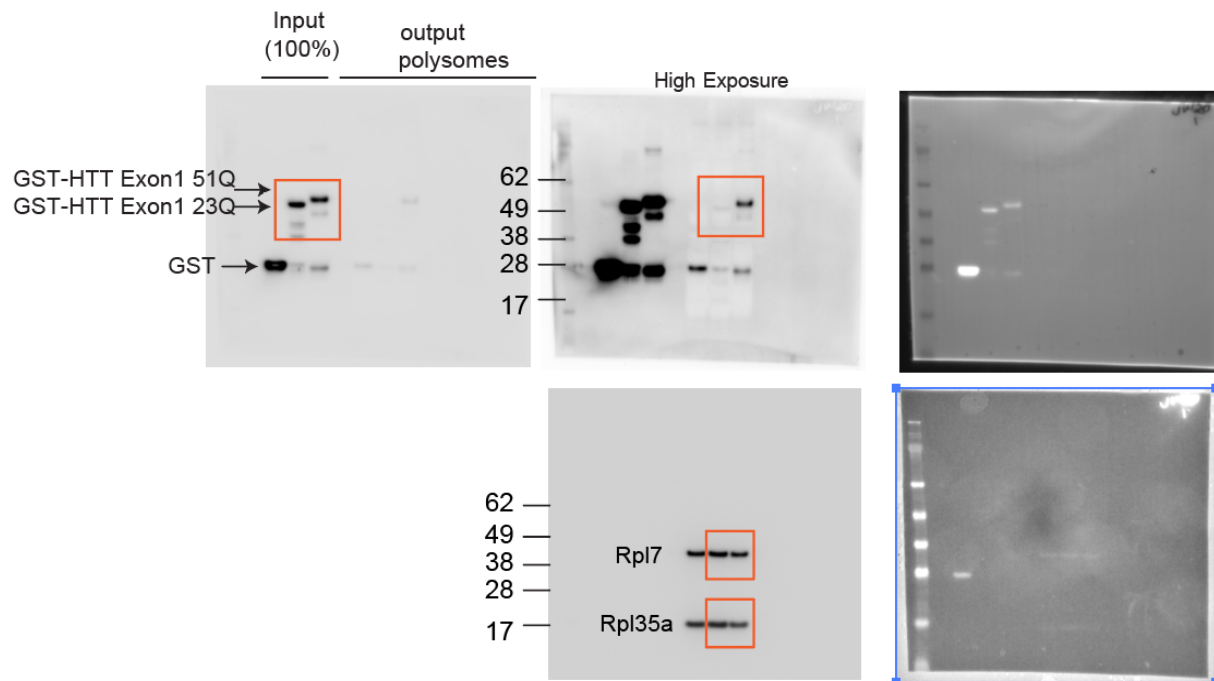

Fig. 9G

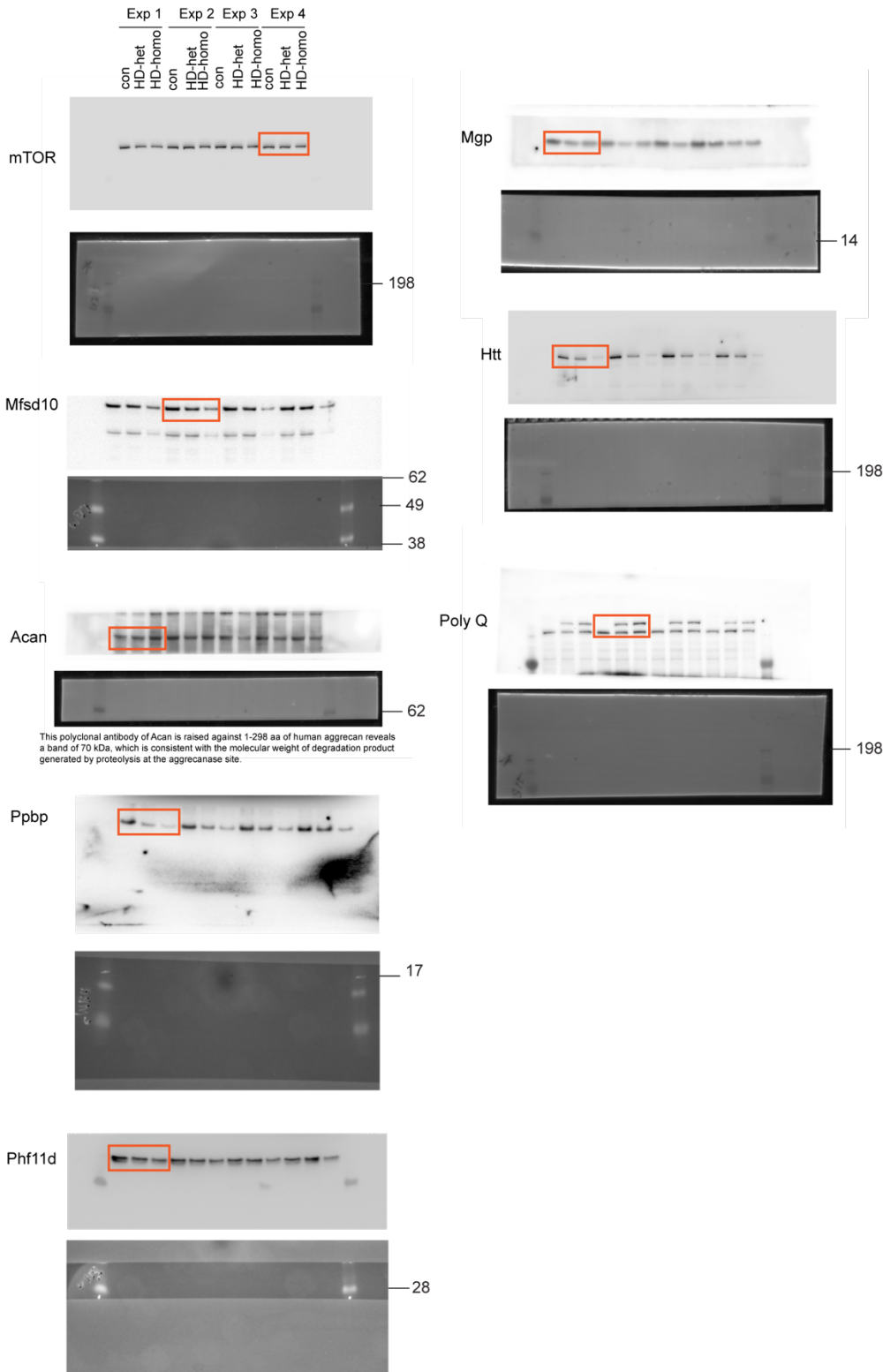

Fig. S2A

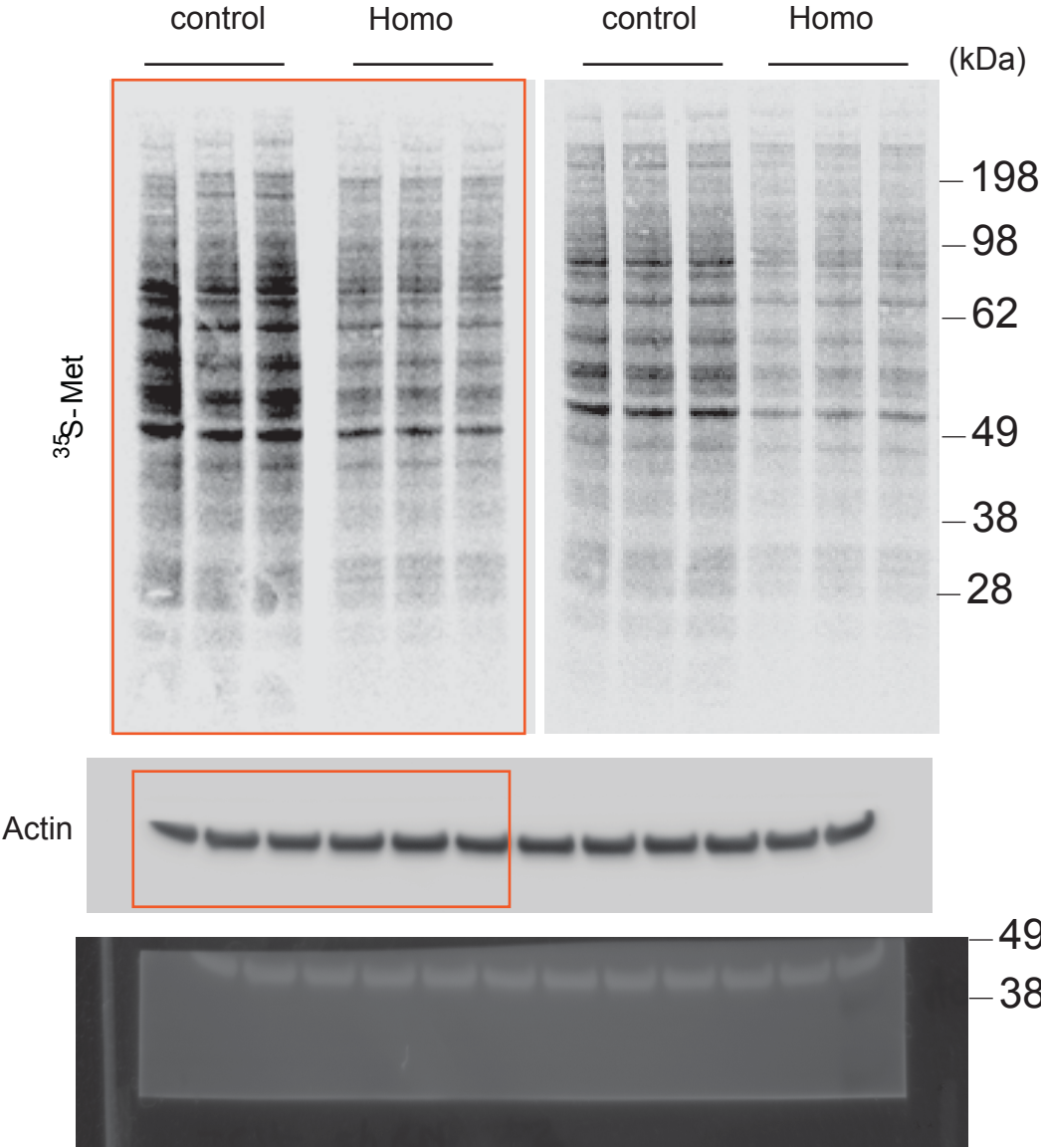

**Fig. S3A**

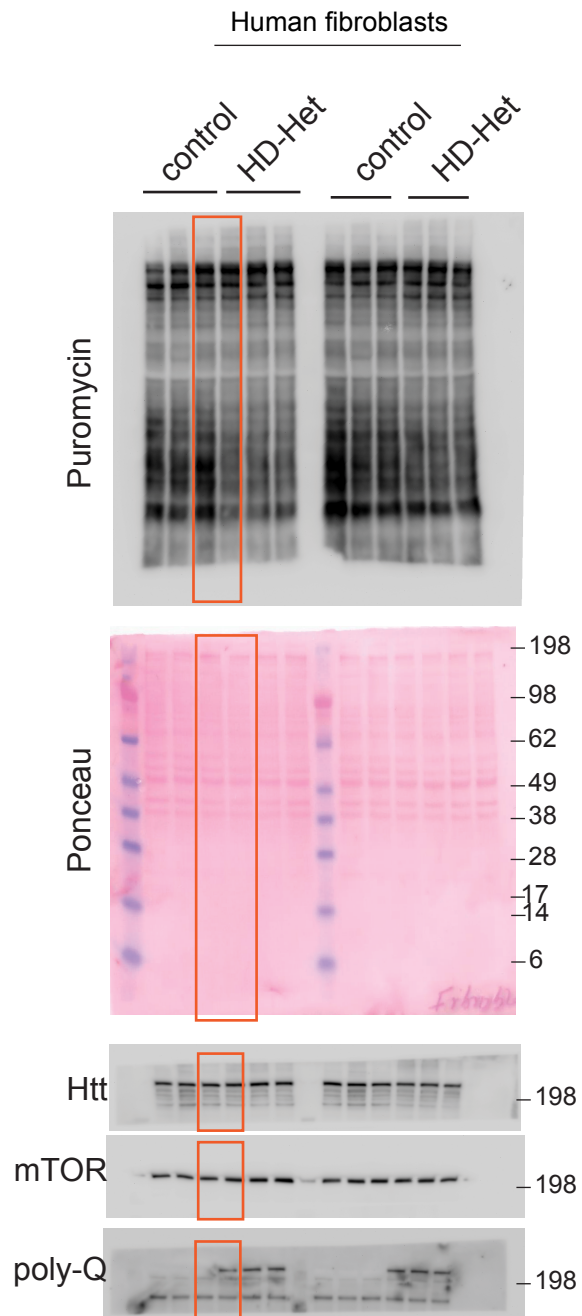

Supplement: Supplementary file 15 — Source Data [file 41467_2021_21637_MOESM15_ESM.zip › Source Raw Figures Compiled_Nat Communication 2021.pdf]
